# Delayed health-seeking behavior and its associated factors among cancer patients in Ethiopia: A systematic review and meta-analysis, 2025

**DOI:** 10.1371/journal.pone.0352869

**Published:** 2026-07-02

**Authors:** Sadik Abdulwehab, Duguma Debela Genati, Frezer Kedir

**Affiliations:** 1 School of Nursing, Wollega University, Oromia, Ethiopia; 2 School of Nursing, Assosa University, Assosa, Ethiopia; 3 School of Nursing, Jimma University, Southwest Oromia, Ethiopia; Haramaya University, ETHIOPIA

## Abstract

**Background:**

Delayed Health-seeking behavior among cancer patients is a major contributor to late diagnosis, poor prognosis, and high mortality, particularly in low-resource settings like Ethiopia. However, evidence on the magnitude and determinants of delayed care-seeking remains fragmented.

**Objective:**

This systematic review and meta-analysis aimed to estimate the pooled prevalence of delayed Health-seeking behavior among cancer patients in Ethiopia and to identify associated factors influencing delays.

**Methods:**

This study employed a systematic review and meta-analysis design to assess delayed Health-seeking behavior and its influencing factors among cancer patients in Ethiopia. A systematic search was conducted in PubMed, Scopus, Web of Science, CINAHL, AJOL, Google Scholar, and Ethiopian University repositories until April 27, 2025. The data were extracted from March 10–20 and analyzed from March 21–30, with report generation till April 27, 2025, using R software. Meta-analysis was performed using a random-effects model, with forest plots illustrating pooled prevalence and associated factors. Heterogeneity was assessed using the I² statistic, and study quality was evaluated using a validated tool.

**Results:**

Seven studies conducted across multiple regions of Ethiopia were included in the final analysis with a total of 2,641 participants. The pooled prevalence of delayed Health-seeking behavior among cancer patients was 54% (95% CI: 39%–68%). Meta-analysis of associated factors showed that rural residence was significantly associated with delayed Health-seeking behavior, with patients residing in rural areas having more than threefold higher odds of delay (AOR = 3; 95% CI: 1.81–4.19), poor knowledge about cancer was strongly associated with delay, with nearly seven times higher odds among patients with poor knowledge compared to those with adequate knowledge (AOR = 6.63; 95% CI: 2.21–11.05), lack of cancer awareness was also a significant predictor of delayed Health-seeking behavior (AOR = 2.63; 95% CI: 1.75–3.51), and patients without pain were over three times more likely to delay Healthcare(AOR = 3.38; 95% CI: 2.44–4.67) were factors associated with delayed Health-seeking behavior.

**Conclusions:**

Our review showed that half of the cancer patients in Ethiopia experienced delayed health-seeking behavior. Delayed care-seeking was associated with rural residence, poor knowledge, limited awareness of cancer, and absence of pain symptoms. Targeted interventions, including public awareness campaigns, expansion of healthcare services in rural areas, and financial support initiatives, are urgently needed to reduce delays and improve early cancer diagnosis and outcomes.

**Prospero Registration Number:**

CRD420251037845

## Introduction

Health-seeking behavior refers to the sequence of actions individuals undertake to recognize symptoms, interpret their severity, and seek appropriate medical care and timely treatment [1]. Delays in seeking care are strongly associated with advanced-stage disease, reduced survival, and increased mortality among cancer patients [2].

Cancer remains a major global public Health challenge, accounting for millions of new cases and approximately 10 million deaths in 2020, making it one of the leading causes of mortality worldwide [3]. In low- and middle-income countries (LMICs), where nearly 70% of cancer-related deaths occur [4,5], 87.2 per 100,000 people die in sub-Saharan Africa (SSA) [6], with an estimated 77,000 new cases and approximately 51,000 deaths annually in Ethiopia [7].

A delayed presentation is particularly pronounced due to structural barriers such as limited diagnostic capacity, weak referral systems, low Health literacy, and sociocultural beliefs, with many patients waiting over three months before seeking care, which is associated with significantly poorer survival outcomes [8–13].

Despite advancements in cancer care, late presentation and diagnosis remain common in sub-Saharan Africa, with evidence showing that approximately 80% of cancer cases are diagnosed at advanced stages [4].

In Ethiopia, despite efforts to expand oncology services, delayed diagnosis remains a critical issue, with more than two-thirds of cancer patients in Ethiopia being diagnosed at advanced stages, largely due to delays in seeking care and systemic barriers [8,9,11,14]. This problem is further compounded by Health-seeking behaviors, as several studies indicate that many patients initially pursue traditional or religious remedies before presenting to formal Healthcare facilities [8,9].

At the policy level, global and national initiatives have emphasized early detection and timely care-seeking as key strategies for reducing cancer mortality [15]. The World Health Organization (WHO) launched the Global Cancer Initiative to promote early detection, timely diagnosis, and effective treatment as core pillars for reducing cancer-related deaths [16–18]. In Ethiopia, the National Cancer Control Plan prioritizes early diagnosis, expansion of oncology services, and public awareness campaigns to address delays in care [19]. However, despite these initiatives, significant gaps remain in implementation, particularly in addressing behavioral, cultural, and socioeconomic barriers influencing Health-seeking behavior [8,9,20].

Existing studies in Ethiopia suggest that delayed Health-seeking behavior is influenced by a complex interplay of socio-demographic, economic, cultural, and Health system factors [10,21–23]. However, the available evidence is fragmented, region-specific, and methodologically heterogeneous, limiting its applicability for national-level decision-making. Moreover, inconsistencies in definitions and measurements of delay further complicate the interpretation of findings across studies. Therefore, a comprehensive synthesis of existing evidence is needed to quantify the magnitude of delayed Health-seeking behavior and to identify its key determinants in the Ethiopian context. Addressing this gap is essential for informing policy decisions, optimizing resource allocation, and designing targeted interventions to improve early cancer detection and patient outcomes. Accordingly, this systematic review and meta-analysis aimed to estimate the pooled prevalence of delayed Health-seeking behavior among cancer patients in Ethiopia and to identify factors significantly associated with delays in seeking care based on available quantitative evidence.

## Methods

### Design

This systematic review and meta-analysis were conducted and reported in accordance with the Preferred Reporting Items for Systematic Reviews and Meta-Analyses (PRISMA) 2020 statement. [24]. A comprehensive search strategy was employed to identify all relevant primary studies conducted in Ethiopia that reported on Health-seeking behavior among cancer patients. Data extraction was carried out using a structured form, and study quality was appraised using standardized tools. Quantitative synthesis using meta-analysis was performed in R software, employing a random-effects model to generate pooled estimates. Statistical heterogeneity and publication bias were assessed, ensuring robust and transparent results.

### Research question

The central question addressed by this review is: What is the prevalence of delayed Health-seeking behavior and the associated factors among cancer patients in Ethiopia?

### Review protocol

To ensure accountability and reduce potential bias, a detailed review protocol was developed before commencing the review. The protocol outlined the review objectives, eligibility criteria, search strategy, data extraction procedures, critical appraisal framework, and synthesis method. It was designed by the Joanna Briggs Institute (JBI) Manual for Evidence Synthesis [25], which provides methodological guidance for qualitative reviews. The protocol was registered with PROSPERO under registration number CRD420251037845 on April 22, 2025, serving as a formal commitment to methodological integrity.

### Search strategy

An exhaustive literature search was conducted across multiple databases, including PubMed, Scopus, Web of Science, Google Scholar, and African Journals Online (AJOL). In addition to database searching, institutional repositories and relevant organizational websites in Ethiopia (such as university repositories and Health-related institutions) were searched manually using predefined keywords, and relevant theses and dissertations were screened based on eligibility criteria to identify potentially relevant studies. Although efforts were made to identify gray literature, no eligible studies meeting the inclusion criteria were found. Keywords were used to capture recently published and non-indexed articles, while MeSH terms ensured the inclusion of studies indexed under standardized subject headings. Boolean operators were applied to combine search terms appropriately and enhance the sensitivity and specificity of the search. The search utilized a combination of keywords and Medical Subject Headings (MeSH): “Health-seeking behavior,” “help-seeking,” “cancer,” “oncology,” “Ethiopia,” “associated factors,” and “delay.” Boolean operators were applied to refine the searches. References of eligible articles were manually screened to identify additional relevant studies. The search was performed through March 10, 2025, and kept up to date until the manuscript submission. Articles were managed using EndNote for duplicate removal. Three independent reviewers screened titles, abstracts, and full texts, resolving disagreements through discussion and consensus. The complete electronic search strategies, including search terms, Boolean operators, and the number of records retrieved from each database, are provided in Table (Table 1).

**Table 1:** Search Strategy and Retrieval Summary for delayed Health-seeking behavior among Cancer Patients in Ethiopia, 2025.

Database	Search Strategy (keywords/MeSH terms)	Records Retrieved	After Duplicates Removed	Full-texts Assessed	Studies Included
PubMed	(“Health-seeking behavior”[MeSH] OR “Health-seeking behavior” OR “help-seeking behavior” OR “delay in care”) AND (“cancer” OR “oncology”) AND “Ethiopia.”	24	18	5	3
Scopus	TITLE-ABS-KEY (“Health seeking behavior” OR “help-seeking behavior” OR “delay in Health care”) AND TITLE-ABS-KEY (“cancer”) AND TITLE-ABS-KEY (“Ethiopia”)	14	10	4	2
Web of Science	(“Health seeking behavior” OR “help-seeking behavior” OR “delay in care”) AND (“cancer”) AND (“Ethiopia”)	12	9	3	1
CINAHL	(MH “Health Seeking Behavior” OR “help-seeking behavior”) AND (MH “Cancer” OR “Oncology”) AND Ethiopia	16	11	2	1
AJOL (African Journals Online)	“Health seeking behavior” AND “cancer” AND “Ethiopia”	9	6	1	1
Google Scholar	Altitle: “Health seeking behavior” AND “cancer” AND “Ethiopia.”	16	12	1	1
Ethiopian University Repositories	“Health seeking behavior” AND “cancer” AND “Ethiopia”	1	1	0	0

### Inclusion and exclusion criteria

Studies were included if they were observational (cross-sectional, case-control, or cohort) and conducted among adult cancer patients in Ethiopia. Eligible studies must have reported on either the prevalence of Health-seeking behavior or examined factors associated with delayed or timely care-seeking. Peer-reviewed publications were considered without restriction on publication year. Excluded studies were reviews, editorials, case reports, conference abstracts, and studies lacking relevant quantitative data or those not conducted in the Ethiopian setting.

### Search outcomes

**Fig 1:**
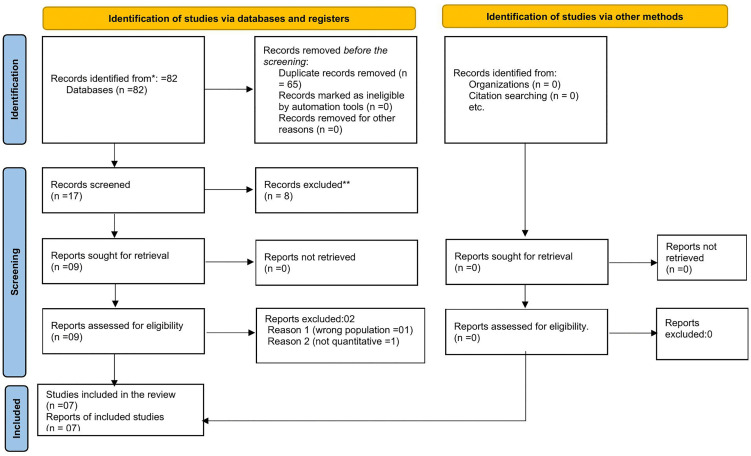
PRISMA Flow diagram describing the selection of studies for the systematic review and meta-analysis on delayed Health-seeking behavior among cancer patients in Ethiopia, 2025. Flow diagram showing the identification, screening, eligibility assessment, and inclusion of studies in the systematic review and meta-analysis of delayed Health-seeking behavior among cancer patients in Ethiopia, 2025.

The database search yielded a total of 82 records: PubMed (07), Scopus (14), Web of Science (12), CINAHL (16), AJOL (9), and Google Scholar (24). After removing duplicates and screening abstracts and full texts, seven studies met the inclusion criteria and were included in the final review. The PRISMA flow diagram summarizes the study selection process, from identification to inclusion (Fig 1).

### Data extraction

Data extraction was conducted using a standardized form adapted from the Joanna Briggs Institute (JBI) guidelines. Three reviewers independently extracted data on study characteristics (author, year, region, design, and population), sample size, and outcomes of interest. Extracted variables included the prevalence of Health-seeking behavior and associated factors. Adjusted odds ratios (AOR), confidence intervals, and p-values were recorded where available. Any disagreements were resolved through discussion or consultation with a third reviewer (Table 2). The data were extracted from March 10–20 and later analyzed from March 21–30, and the report generation was completed by April 27, 2025.

**Table 2:** Characteristics of included studies on delayed Health-seeking behaviors and associated factors among Cancer Patients in Ethiopia, 2025.

Author & Year	Title	Region/Setting	Design	Sample & Population	Prevalence with CI	Operational Definition of Delay	Associated Factors (AOR, CI, P-value)	Limitations	Conclusions	Recommendations
Bayable et al., 2023	Delay in Health-seeking behaviour and associated factors among adult patients with cancer in Ethiopia	Ethiopia (TASH and Felege Hiwot)	Cross-sectional	407 adult cancer patients	50.4% (95% CI: 45.7–55.3)	≥3 months from symptom recognition to first Healthcare visit	- Rural residence (AOR = 2.19; 95% CI: 1.29–3.72)- Lack of knowledge (AOR = 2.62; 95% CI: 1.63–4.23)- No Health insurance (AOR = 2.76; 95% CI: 1.46–5.24)- No family support (AOR = 2.34; 95% CI: 1.38–3.98)- Visited a traditional healer (AOR = 2.07; 95% CI: 1.21–3.55)	Recall and social desirability bias	Half delayed seeking care; modifiable factors identified	Increase awareness, insurance coverage, and support services
Berhane et al., 2019	Determinants of delayed diagnosis among pediatric cancer patients from Ayder Comprehensive Specialized Hospital, Tigray, Ethiopia	Tigray Region (Ayder Hospital)	Cross-sectional	102 pediatric cancer patients	75.5% (CI not specified)	>90 days (above 3rd quartile) from symptom onset to confirmed diagnosis (includes patient + physician delay)	- Rural residence (AOR = 3.1; 95% CI: 1.1–8.9)- Caregivers with no formal education (AOR = 4.5; 95% CI: 1.4–14.3)- Distance >10 km to Health facility (AOR = 4.2; 95% CI: 1.3–13.5)	Small sample, single setting	High diagnostic delay; education and geography matter	Improve rural access and caregiver education
Habtu et al., 2018	Health-seeking behavior and its determinants for cervical cancer among women of childbearing age in Hossana town, Ethiopia	Hossana town, Ethiopia	Community-based cross-sectional	583 women of childbearing age	17.3% (CI not reported)	>3 months from first symptom recognition to first Healthcare visit	- Good knowledge (AOR = 2.0; 95% CI: 1.13–3.56)- Positive attitude (AOR = 3.3; 95% CI: 1.96–5.59)- Family history of cervical cancer (AOR = 2.6; 95% CI: 1.31–5.26)	Social desirability bias, cross-sectional	Health-seeking is low despite awareness	Promote education and early screening efforts
Hassen et al., 2021	Factors Associated with Delay in Breast Cancer Presentation at the Only Oncology Center in North East Ethiopia	Dessie Referral Hospital, North East Ethiopia	Institution-based cross-sectional study	204 women with pathologically confirmed breast cancer (18 + years)	50.5% delayed (>3 months); Median delay: 4 months	Preventive Health-seeking behavior: intention to be screened before symptoms (not diagnostic delay)	- Age ≥ 40: AOR = 4.81 (1.26–18.65), p < 0.021- College + : AOR = 0.05 (0.01–0.77), p < 0.031- Employed: AOR = 0.14 (0.03–0.91), p < 0.002- Urban residence: AOR = 0.21 (0.01–0.82), p < 0.001- Visited traditional healer: AOR = 0.38 (0.2–0.69), p < 0.0037- No lump in under armpit: AOR = 9.05 (1.14–22.69), p < 0.037	causality- Recall bias, small sample size for broader generalization	Delayed presentation was high. Older age, illiteracy, unemployment, rural residence, and cultural practices (traditional healing) are significant predictors.	Interventions to improve early detection, Health literacy, and address cultural/traditional beliefs are necessary. Enhance awareness and access in rural settings.
Kussia et al., 2024	Health care seeking behaviour towards cervical cancer screening among women aged 30–49 years in Arba Minch town, Southern Ethiopia, 2023	Arba Minch Town, Southern Ethiopia	Community-based cross-sectional study	414 women aged 30–49 years	47.6% [95% CI: 42.7–52.5%]	Preventive Health-seeking behavior: uptake of cervical cancer screening (does not report the exact delay of the time interval)	- Good knowledge (AOR = 1.55; 95% CI: 1.01–2.39; *p* < 0.05) - Positive perceived susceptibility (AOR = 3.63; 95% CI: 2.06–6.42; *p* < 0.001) - Positive perceived severity (AOR = 2.65; 95% CI: 1.71–4.09; *p* < 0.001) - Positive perceived benefits (AOR = 4.85; 95% CI: 2.92–7.87; *p* < 0.001)	social desirability bias causal inference - The Health Belief Model has known limitations in predicting behavior alone	The prevalence of Health care-seeking behavior towards cervical cancer screening was low. Factors such as knowledge, perceived susceptibility, perceived severity, and perceived benefits were significantly associated.	Programs should enhance women’s awareness, address perceived benefits and severity, and improve risk perception to increase screening behaviors. Targeted behavioral change communication strategies are recommended.
Legese et al., 2021	Information Needs Among Breast Cancer Patients Attending Care at TASH	TASH, Addis Ababa, Ethiopia	Cross-sectional	N = 375 breast cancer patients	High information needs; overall mean = 238.7 (SD = 22.5)	Not report (study focused on information needs, and as their information was not given and delayed)	One-way ANOVA used; detailed AORs not reported in the excerpt	Study limited to one tertiary hospital; cross-sectional design limits causal inference	High unmet information needs in all five subscales (treatment, disease, tests, physical, psychosocial)	Provide targeted Health education and tailored information provision across all stages of breast cancer care
Mekuria et al., 2016	Preferred information sources and needs of cancer patients on disease symptoms and management	Gondar University Referral Hospital and Tikur Anbesa Specialized Hospital, Ethiopia	Cross-sectional study	556 adult cancer patients undergoing chemotherapy	67.26% wanted information on a specific type of cancer, 63.29% on chemo side effects, 51.8% on prognosis;	Not report (study focused on information sources as they wanted to get Health-seeking information, and the information was delayed)	Information needs varied based on demographics and treatment stage (not quantified with AORs in this paper)	Data collected only from two hospitals, limiting generalizability; reliance on self-report may introduce bias	Patients reported high unmet need for information, especially on disease specifics, treatment side effects, and prognosis.	Improve communication from Healthcare providers beyond doctors (e.g., clinical pharmacists). Regular assessment of patient information needs.

We observed heterogeneity in the operational definitions of ‘delay’ across included studies, with thresholds ranging from ≥3 months after symptom recognition to >90 days before diagnosis confirmation. In some studies, preventive Health-seeking behavior rather than diagnostic delay was reported. To account for this conceptual variability, we extracted and reported each study’s definition in Table 2 and applied a random-effects model in the meta-analysis, which allows for between-study heterogeneity in effect estimates. While these do not measure delay directly, they provide contextual insights into barriers and facilitators of timely care and showed that they didn’t get Health information on time, and the patients wanted to get it, which contributes indirectly to delayed diagnosis and Health-seeking information.

### Organizing, summarizing, and reporting the results

Findings were organized into three main categories: Demographic and clinical characteristics of cancer patients experiencing delayed Health-seeking behavior, and Factors associated with delayed Health-seeking behavior. Meta-analyses were conducted to pool estimates of the prevalence of delayed Health-seeking behavior and its significant associated factors. Results were presented in descriptive and tabular formats and illustrated using charts and figures. The PRISMA 2020 checklist guided the reporting process to ensure rigor, transparency, and reproducibility.

### Quality appraisal

Following data extraction, each included study was critically appraised for methodological quality using the Joanna Briggs Institute (JBI) Critical Appraisal Checklist for Analytical Cross-Sectional Studies [25]. Three independent reviewers evaluated each study based on key criteria, including sampling methods, outcome measurement, confounding control, and appropriateness of statistical analysis. Studies were scored out of eight “Yes” responses: scores of 7–8 were considered low risk of bias, 4–6 moderate risk, and below 4 high risk. Only studies rated as low or moderate risk were included in the final synthesis. Any disagreements among reviewers were resolved through discussion or consultation with a third reviewer. This rigorous appraisal ensured that the findings on delayed Health-seeking behavior were based on methodologically sound evidence (Table 3).

**Table 3:** JBI Critical Appraisal on delay Health-seeking behaviors among cancer patients in Ethiopia, 2025.

Study No.	Bayable et al., 2023	Berhane et al., 2019	Habtu et al., 2018	Hassen et al., 2021	Kussia et al., 2024	Legese et al., 2021	Mekuria et al., 2016
Was there a clear statement of the aims of the research?	✔	✔	✔	✔	✔	✔	✔
Was the study design appropriate for the aims of the research?	✔	✔	✔	✔	✔	✔	✔
Was the sample representative of the population studied?	✔	✘	✔	✔	✔	✘	✔
Was the sample size adequate?	✔	✘	✔	✔	✔	✔	✔
Were the study subjects and the setting described in detail?	✘	✔	✔	✔	✔	✔	✔
Was the data collected reliably?	✔	✔	✔	✘	✔	✔	✔
Were the statistical analyses used to assess the data appropriate?	✔	✔	✔	✔	✔	✘	✘
*Were the findings valid and applicable to the local context?*	✔	✔	✔	✔	✔	✔	✔
*Overall Quality*	7	6	8	7	8	6	7

Key: ✔ = Yes (Criterion met), ✘ = No (Criterion not met) and? = Unclear (Not adequately reported)

### Statistical analysis

Data synthesis was performed using R software. Descriptive statistics (frequencies, means, and standard deviations) were used to summarize study characteristics. Meta-analyses employed a random-effects model due to anticipated heterogeneity across studies. Pooled prevalence estimates and pooled odds ratios for significant associated factors were calculated, each with 95% confidence intervals. Heterogeneity was assessed using the I² statistic, with I² > 50% considered substantial. Publication bias was evaluated using Egger’s test and visual inspection of funnel plots. Sensitivity analyses were conducted by excluding studies with small sample sizes or high bias.

### Ethical consideration

This study involved a secondary analysis of previously published research and did not require formal ethical approval. All efforts were made to ensure ethical integrity by including only studies that had obtained ethical clearance and informed consent from participants. Intellectual property was respected through appropriate citation and acknowledgment of original authors.

## Result

### Characteristics of the included studies

The included studies were conducted in various regions of Ethiopia, encompassing both hospital-based and community-based settings. Hospital-based studies were conducted at Tikur Anbessa Specialized Hospital [8,26], Felege Hiwot Referral Hospital [8], Ayder Comprehensive Specialized Hospital [27], Dessie Referral Hospital [28], and Gondar University Referral Hospital [29]. Community-based studies were undertaken in Hossana Town [30] and Arba Minch Town [31] (Table 2).

All included studies employed a cross-sectional design. Institution-based studies were conducted by Bayable et al.[8], Berhane et al. [27], Hassen et al. [28], Legese et al. [26], and Mekuria et al. [29], while Habtu et al. [30] and Kussia et al. [31] used community-based cross-sectional designs to assess Health-seeking behaviors and associated factors among cancer patients.

Sample sizes across the studies ranged from 102 to 583 participants [27,30]. Most studies focused on adult cancer patients [8,26,28–31], while Berhane et al. [27] focused on pediatric cancer patients.

### Prevalence of delayed Health-seeking behavior among cancer patients

**Fig 2:**
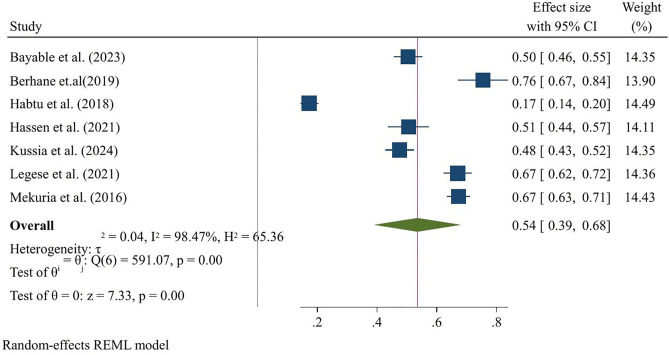
Forest plot of the prevalence of delayed Health-seeking behavior among cancer patients in Ethiopia,2025. Studies are presented by first author and publication year, with corresponding prevalence estimates and 95% confidence intervals (CIs). Square markers indicate study-specific estimates, with size proportional to study weight, and horizontal lines represent 95% CIs. The diamond indicates the pooled prevalence estimate (54%; 95% CI: 39%–68%).

The overall prevalence of delayed Health-seeking behavior among cancer patients in Ethiopia, based on the included studies, varied widely. The prevalence rates ranged from 17.3% to 75.5% [3–9]. The pooled prevalence of delayed Health-seeking behavior among cancer patients was 54% (CI: 39–68) (Fig 2).

The heterogeneity analysis revealed substantial variability among the included studies. The between-study variance was estimated at τ² = 0.04, indicating differences in effect sizes across studies. The I² statistic was 98.47%, demonstrating very high heterogeneity, while the Cochran’s Q test was statistically significant (Q = 591.07, p < 0.001), confirming the presence of considerable between-study variation. Due to this high level of heterogeneity, the pooled prevalence estimate should be interpreted with caution, as the observed variation may reflect differences in study populations, settings, methodologies, and operational definitions across the included studies. Therefore, a random-effects REML model was employed to account for between-study variability and provide a more conservative pooled estimate.

**Fig 3:**
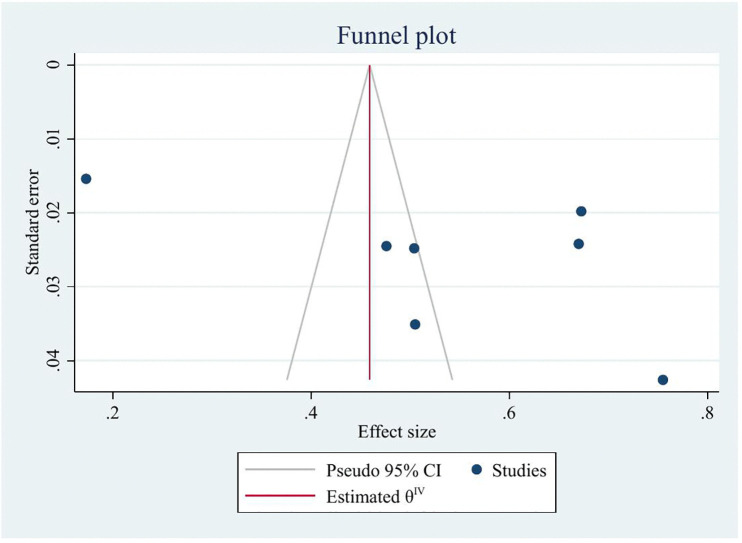
Funnel plot for assessment of publication bias in studies of delayed Health-seeking behavior among cancer patients in Ethiopia,2025. Each point represents an individual study plotted by effect size and standard error. The vertical line indicates the pooled estimate, and the diagonal lines represent the 95% confidence limits.

A funnel plot with pseudo 95% confidence limits was constructed to evaluate potential publication bias. Although a slight asymmetry was observed in the distribution of studies around the pooled effect estimate, there was no clear indication of substantial publication bias. Considering the limited number of included studies, the funnel plot should be interpreted cautiously. These findings, together with the results of Egger’s regression test, suggest that publication bias is unlikely to have materially influenced the pooled estimate (Fig 3).

### Sensitivity analysis

**Fig 4:**
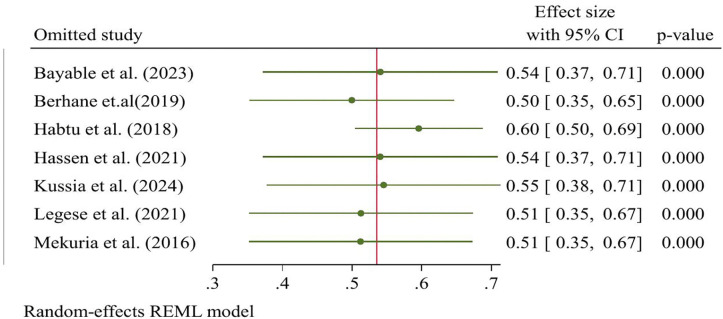
Forest plot of the sensitivity analysis for the pooled prevalence of delayed health‑seeking behavior among adult cancer patients in Ethiopia, 2025. Studies are presented by first author and publication year, with corresponding prevalence estimates and 95% confidence intervals (CIs). Square markers represent study‑specific estimates, with marker size proportional to study weight, and horizontal lines indicate the 95% CIs.

The study conducted a sensitivity analysis using a random-effects model to determine the impact of a single study on the overall meta-analysis. The results showed no strong evidence for a single study’s influence on the overall meta-analysis. The estimates from a single study were closer to the combined estimate, indicating no single-study effect. The analysis also showed that removing a single study did not significantly affect the pooled estimate, indicating the robustness of the findings (Fig 4).

To explore potential age-related heterogeneity, we conducted a sensitivity analysis excluding the single pediatric study [27]. To examine the influence of age group, we conducted a sensitivity analysis excluding the single pediatric study [27]. The pooled prevalence estimate without the pediatric study was 50% (95% CI:35–65), compared to 54% (95% CI:39–68) when all seven studies were included. This small difference indicates that the inclusion or exclusion of the pediatric study did not materially alter the overall pooled estimate. Given that only one pediatric study was available, a formal subgroup meta-analysis was not statistically robust; therefore, results are reported narratively.

### Factors associated with delayed Health-seeking behavior among cancer patients

Two studies found that age was significantly associated with delayed Health-seeking behavior among cancer patients [27,28]. Patients aged 5–10 years were 2.19 times more likely to delay seeking Healthcare compared to those under 5 years old (OR = 2.19; 95% CI: 1.00–7.23) [27]. Similarly, individuals aged over 10 years were 4.01 times more likely to delay seeking care compared to those under 5 years old (OR = 4.01; 95% CI: 1.55–12.00) [27]. Additionally, patients aged 40 years or older were found to be 4.81 times more likely to delay Health-seeking Healthcare compared to their younger counterparts (OR = 4.81; 95% CI: 1.26–18.65) [28]. Three studies demonstrated a positive association; however, due to differences in variable categorization and analytical adjustments, meta-analysis was not performed. Instead, findings were synthesized narratively.

**Fig 5:**
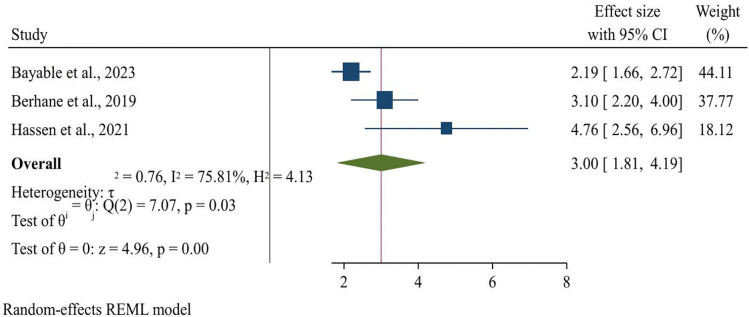
Forest plot of the association between residence (rural vs. urban) and delayed Health-seeking behavior among cancer patients in Ethiopia,2025. Studies are presented by first author and publication year, with odds ratios (ORs) and 95% confidence intervals (CIs). Square markers indicate study-specific estimates, with size proportional to study weight, and horizontal lines represent 95% CIs. The diamond indicates the pooled effect estimate.

The reviewed article showed that the Residence area showed a significant association with delayed Health-seeking behavior among cancer patients in Ethiopia. Rural residence was consistently identified as a risk factor across multiple studies [8,27,28]. The pooled adjusted odds ratio (AOR) for the association between rural residence and delayed Health-seeking behavior was 3 (95% CI: 1.81–4.19), indicating that individuals living in rural areas were more than three times as likely to experience delays in seeking Healthcare compared to urban residents (Fig 5).

The heterogeneity analysis indicated substantial variability among the included studies. The between-study variance was estimated at τ² = 0.76, suggesting considerable differences in effect sizes across studies. The I² statistic was 75.81%, indicating substantial heterogeneity, while the Cochran’s Q test was statistically significant (Q = 7.07, p = 0.03), confirming the presence of significant between-study variation. Due to this level of heterogeneity, the pooled effect estimate should be interpreted with caution, as differences in study populations, settings, and methodological characteristics may have contributed to the observed variability. Therefore, a random-effects REML model was used to account for between-study heterogeneity and generate a more robust pooled estimate.

**Fig 6:**
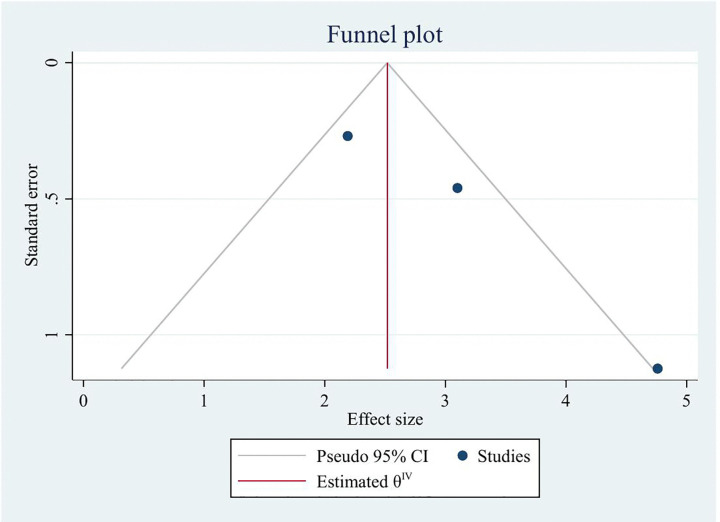
Funnel plot for assessment of publication bias in studies examining the association between residence and delayed Health-seeking behavior among cancer patients in Ethiopia,2025. Each point represents an individual study plotted by effect size and standard error. The vertical line indicates the pooled estimate, and the diagonal lines represent the 95% confidence limits.

A funnel plot with pseudo 95% confidence shows slight asymmetry was noted in the distribution of studies around the pooled effect estimate; there was no strong evidence of substantial bias. Given the limited number of included studies, the funnel plot should be interpreted with caution. Taken together with the results of Egger’s regression test, these findings suggest that publication bias is unlikely to have materially influenced the pooled estimate (Fig 6).

**Fig 7:**
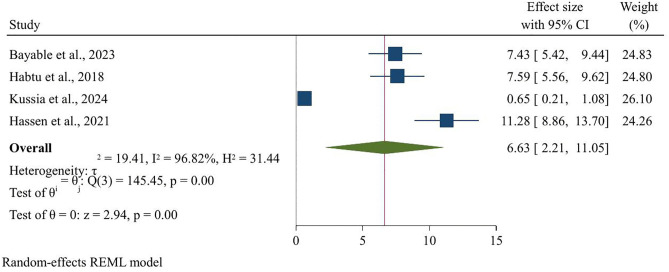
Forest plot of the association between knowledge status and delayed Health-seeking behavior among cancer patients in Ethiopia, 2025. Studies are presented by first author and publication year, with odds ratios (ORs) and 95% confidence intervals (CIs). Square markers indicate study-specific estimates, with size proportional to study weight, and horizontal lines represent 95% CIs. The diamond indicates the pooled effect estimate.

Four studies showed that Knowledge status was found to be a significant factor associated with delayed Health-seeking behavior among cancer patients in Ethiopia [8,28,30,31]. Patients with poor knowledge about cancer and its symptoms had significantly higher odds of delaying care compared to those with better knowledge. The pooled adjusted odds ratio (AOR) from the meta-analysis showed that poor knowledge was associated with nearly seven times higher odds of delayed Health-seeking behavior (AOR = 6.63, 95% CI: 2.21–11.05), indicating a strong and statistically significant association (p < 0.001) (Fig 7).

The heterogeneity analysis demonstrated very high inconsistency among studies, with I² = 96.82% and χ² = 19.41 (p < 0.001), suggesting that nearly all observed variation was due to genuine differences rather than chance. The H² value of 31.44 further confirmed the magnitude of heterogeneity. These findings highlight considerable between‑study variance, warranting cautious interpretation of the pooled estimate and emphasizing the importance of contextual factors in explaining differences across study results.

**Fig 8:**
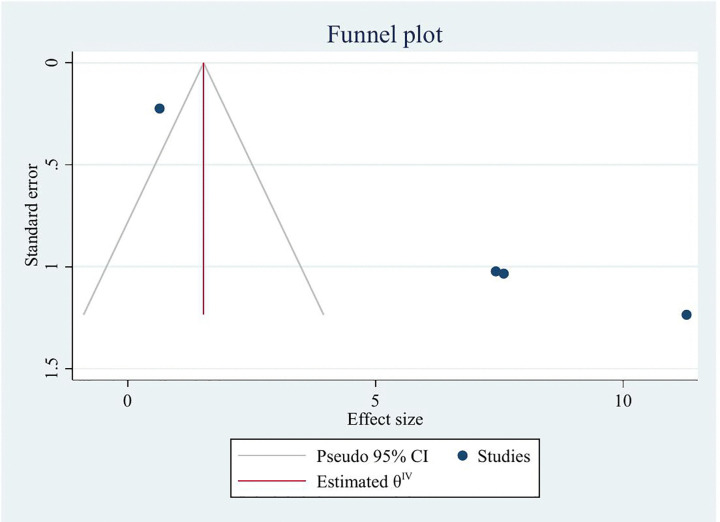
Funnel plot for assessment of publication bias in studies examining the association between knowledge status and delayed Health-seeking behavior among cancer patients in Ethiopia, 2025. Each point represents an individual study plotted by effect size and standard error. The vertical line indicates the pooled estimate, and the diagonal lines represent the 95% confidence limits.

A funnel plot with pseudo 95% confidence limits was constructed to evaluate potential publication bias. The distribution of studies around the pooled effect estimate appears relatively balanced, though minor asymmetry can be observed. Most study points fall within the expected confidence boundaries, suggesting no strong evidence of substantial publication bias. Given the limited number of included studies, the visual interpretation should be made cautiously (Fig 8).

**Fig 9:**
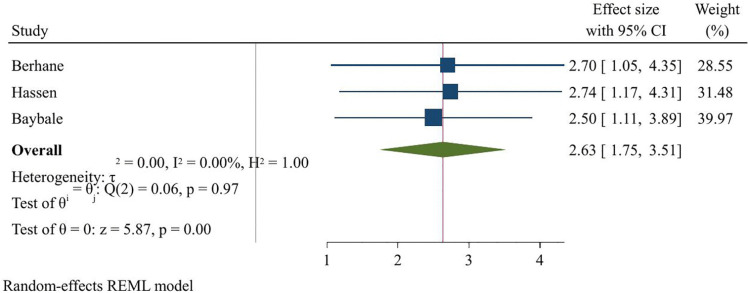
Forest plot of the association between cancer awareness and delayed Health-seeking behavior among cancer patients in Ethiopia,2025. Studies are presented by first author and publication year, with odds ratios (ORs) and 95% confidence intervals (CIs). Square markers indicate study-specific estimates, with size proportional to study weight, and horizontal lines represent 95% CIs. The diamond indicates the pooled effect estimate.

Three studies showed that there is an association between cancer awareness/knowledge and delayed Health-seeking behavior among cancer patients [8,27,28]. The pooled adjusted odds ratio (AOR) for the association between lack of cancer awareness and delayed Health-seeking behavior was 2.63 (95% CI: 1.75–3.51), indicating that individuals without cancer awareness were more than two times as likely to delay seeking Healthcare compared to those with some level of awareness (Fig 9).

The analysis showed no significant heterogeneity among the included studies, with a Tau² of 0 and an I² value of 0%, suggesting that any variability in the results was due to chance, not true differences between the studies. The Q statistic (0.06, p = 0.97) further confirmed the homogeneity of the studies.

**Fig 10:**
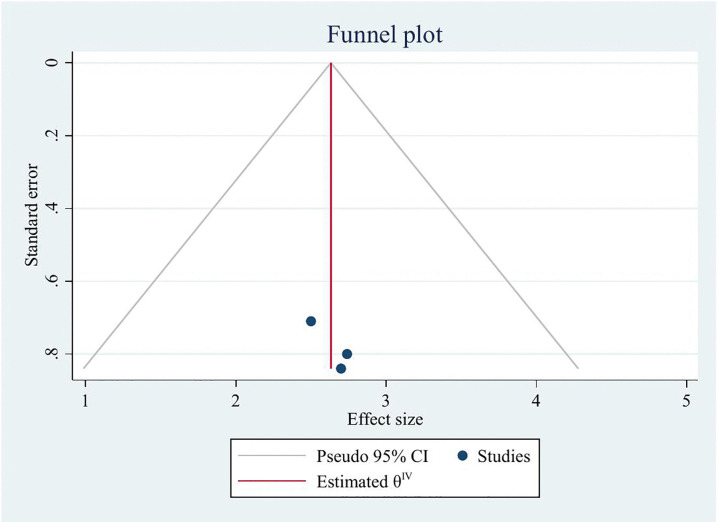
Funnel plot for assessment of publication bias in studies examining the association between cancer awareness and delayed Health-seeking behavior among cancer patients in Ethiopia,2025. Each point represents an individual study plotted by effect size and standard error. The vertical line indicates the pooled estimate, and the diagonal lines represent the 95% confidence limits.

A funnel plot with pseudo 95% confidence limits was constructed to assess the presence of publication bias among the included studies. The distribution of study points around the pooled effect estimate (red vertical line) shows a generally balanced pattern, though minor asymmetry is visible. Most studies fall within the expected confidence boundaries, suggesting that substantial publication bias is unlikely. However, given the relatively small number of studies, the visual interpretation should be approached with caution (Fig 10).

A meta-analysis of two studies examining the association between pain and delayed Healthcare-seeking among cancer patients showed that individuals with no pain were significantly more likely to delay seeking Healthcare compared to those experiencing pain [8,28]. Hassen’s study found that individuals with no pain were 2.28 times more likely to delay Healthcare compared to those experiencing pain (OR = 2.28; 95% CI: 1.26–4.13) [28]. Similarly, Baybale’s study found that individuals with no pain were 4.55 times more likely to delay Healthcare compared to those with pain (OR = 4.55; 95% CI: 3.03–6.66) [8]. The pooled odds ratio was 3.38 (95% CI: 2.44–4.67), indicating that patients without pain were over three times more likely to delay Healthcare.

Several factors were identified as significantly associated with delayed Health-seeking behavior in individual studies, but could not be pooled due to being reported by a single study. These factors included marital status, social support, absence of pain symptoms, and visiting traditional or spiritual healers.

The study by Hassen found that visiting a traditional/spiritual healer was significantly associated with delayed Health-seeking behavior among cancer patients [28]. Patients who visited a traditional/spiritual healer were 2.63 times more likely to delay seeking Healthcare compared to those who had not visited a traditional healer (OR = 2.63; 95% CI: 1.5–5.0, p = 0.002).

One study found that marital status was significantly associated with delayed Health-seeking behavior among cancer patients. Individuals who were married were 2.51 times more likely to delay seeking Healthcare compared to those who were single (OR = 2.51; 95% CI: 0.65–9.62) [28].

Again, one study found that social support was significantly associated with delayed Health-seeking behavior among cancer patients [8]. Patients with low social support were 9.7 times more likely to delay seeking Healthcare compared to those with high social support (OR = 9.7; 95% CI: 6.38–14.91). Similarly, those with moderate social support were 3.2 times more likely to delay Healthcare-seeking compared to patients with high social support (OR = 3.20; 95% CI: 1.72–5.93). Pooling of results was not conducted, as the categorization of social support varied across studies, preventing direct comparison and synthesis.

Two studies found that financial status was significantly associated with delayed Health-seeking behavior among cancer patients [27,28]. Patients with a monthly income of less than 1000 ETB were 6.1 times more likely to delay seeking Healthcare compared to those with an income greater than 3000 ETB (OR = 6.1; 95% CI: 1.76–7.23) [27]. Similarly, patients with a monthly income of more than 3001 ETB were 0.38 times less likely to delay seeking care compared to those with an income of 1250 ETB or less (OR = 0.38; 95% CI: 0.17–0.86) [28]. Pooling of results was not conducted, as the categorization of income varied across studies, preventing direct comparison and synthesis. Additionally, individuals without Health insurance were 2.4 times more likely to delay seeking Healthcare compared to those with Health insurance (OR = 2.4; 95% CI: 1.50–3.50) [27].

## Discussion

This systematic review included seven studies conducted across different regions of Ethiopia, both in hospital and community settings [8,26–31]. The review showed that the overall pooled prevalence of delayed Health-seeking behavior among cancer patients in Ethiopia was 52%(CI: 36–68), which is higher than the study done in Taiwan, which accounts for 25.2% [32], in Ghana accounts for 34.4% [33], and in India accounts 34.2% [34]. This discrepancy may be explained by differences in Health system capacity, socioeconomic conditions, and sociocultural influences. Drawing on the Health Belief Model, delayed care in Ethiopia may be driven by greater perceived barriers such as limited access to Healthcare services, financial constraints, low cancer awareness, and reliance on traditional healing practices [35]. Additionally, rural–urban disparities and weak referral systems further contribute to delays. These findings have important clinical and public Health implications, as delayed Health-seeking is associated with late-stage diagnosis and poorer outcomes. Therefore, strengthening early detection services, improving Healthcare accessibility, and implementing culturally appropriate awareness interventions are essential to reduce delays and improve cancer outcomes in Ethiopia.

Our findings primarily reflect adult cancer populations, with only one included study focusing exclusively on pediatric patients [27]. Sensitivity analysis demonstrated that excluding this pediatric study yielded a pooled prevalence estimate of 49%, compared to 52% when all six studies were included. This small difference indicates that the overall pooled estimate was not materially influenced by the pediatric data. Nevertheless, the pediatric study highlighted unique determinants of delay that differ from the adult-focused predictors reported elsewhere. These differences suggest that age-related heterogeneity in Health-seeking behavior is likely, but remains underexplored in Ethiopia. Future research should therefore prioritize age-stratified analyses, ideally with larger pediatric cohorts, to better capture the distinct barriers and facilitators influencing timely cancer diagnosis across the life course.

Three studies [8,27,28] measured diagnostic delay using thresholds of ≥3 months or >90 days. Other studies [31] assessed preventive Health-seeking behavior for cervical cancer screening, while Legese et al. and Mekuria et al. explored information needs. Although conceptually distinct, these findings highlight overlapping determinants such as poor knowledge, low awareness, and reliance on alternative sources, which contribute indirectly to delayed diagnosis.

This review showed that there is an inverse relationship between age and delayed Health-seeking behavior among cancer patients [27,28], which is similar to studies done in Nigeria [36], Ghana [33], and China [37]. This pattern may be explained by factors outlined in the Health Belief Model, where older individuals may face greater perceived barriers, such as limited symptom awareness, fear of diagnosis, financial constraints, and cultural influences that discourage timely care-seeking [35]. These findings highlight important clinical and public Health implications, emphasizing the need for targeted interventions that improve awareness, enhance access to affordable Healthcare services, and address sociocultural and economic barriers, particularly among older populations, to promote early diagnosis and better cancer outcomes.

The reviewed article showed that the Residence area showed a significant association with delayed Health-seeking behavior among cancer patients in Ethiopia, as residing in rural areas was consistently identified as a risk factor across multiple studies [8,27,28]. This finding similar with study done in Ghana [33],Japan [38], Uganda [39], Scotland [40], and USA [41]. This may be due to limited Healthcare access, poor transportation, low Health literacy, financial constraints, and reliance on traditional medicine in rural areas. These findings highlight the need to strengthen rural Healthcare services, improve access and referral systems, and enhance community-based cancer awareness to reduce delays and improve outcomes.

A reviewed article indicates that marital status can influence the timeliness of Health-seeking behavior among cancer patients, as married individuals were more likely to delay seeking Healthcare compared to single individuals. This may be attributed to factors such as prioritizing family responsibilities, financial constraints, or reliance on spousal support, which can inadvertently lead to the postponement of medical consultations. Conversely, a study done in China [42], Italy [43], and the USA [44] highlighted that unmarried, widowed, or divorced patients often present with more advanced stages of cancer, suggesting that the absence of a partner may lead to reduced encouragement to seek prompt medical attention and lower Health awareness. These contrasting findings underscore the complex role of marital status in Health-seeking behaviors, emphasizing the need for targeted interventions that consider the unique challenges faced by individuals based on their marital status.

A study reveals that social support significantly influences delayed Health-seeking behavior among cancer patients, with low social support patients being more likely to delay Healthcare-seeking [8]. The findings align with studies done in Ethiopia [45], Denmark [46], the USA [47,48], and the UK [49]. This may be explained by the role of social networks in facilitating symptom recognition, decision-making, and access to care, where a lack of support can delay timely medical consultation and worsen outcomes. These findings highlight the need to integrate psychosocial support, such as counseling and patient support groups, into cancer care, and to strengthen public Health efforts that promote the importance of social support in encouraging timely Healthcare-seeking.

Two reviewed articles showed that cancer patients with lower monthly incomes were more likely to delay Healthcare seeking [27,28].the finding similar with study done in Sub-Saharan Africa [50],USA [51], Maryland [52],New York [53]. This may be due to financial barriers such as treatment costs, lack of insurance, and transportation challenges, which limit timely access to care. These findings highlight the need to strengthen financial support systems and improve access to affordable cancer services. However, the evidence is based on a limited number of studies with varying income measures, although consistent findings across settings support the reliability of this association.

The study showed that individuals without Health insurance were 2.4 times more likely to delay seeking Healthcare compared to those with Health insurance [27], which is similar to a study finding in Ghana [54], the USA [55], Mexico [56], and the Philippines [57]. This association can be explained by financial and access barriers, where a lack of insurance increases out-of-pocket costs and discourages timely care-seeking. These findings emphasize the need to expand insurance coverage and reduce financial barriers to improve early Healthcare utilization. However, this evidence is based on a limited number of studies, although consistency across settings supports the robustness of the finding.

Four studies showed that Knowledge status was found to be a significant factor associated with delayed Health-seeking behavior among cancer patients in Ethiopia [8,28,30,31], as patients with poor knowledge about cancer and its symptoms had significantly higher odds of delaying care compared to those with better knowledge, which is similar to a study done in Ghana [33,58], Uganda, and South Africa [59]. This is due to cancer patients’ lack of awareness about symptoms and disease stages, which are causing Healthcare delays, advanced disease stages, and poor treatment outcomes. Public Health campaigns targeting misconceptions, early detection, and clear cancer symptoms are needed.

Three studies showed that there is an association between cancer awareness/knowledge and delayed Health-seeking behavior among cancer patients [8,27,28], suggesting that individuals without cancer awareness were more than two times as likely to delay seeking Healthcare compared to those who had some level of cancer awareness. The finding is similar to a study done in Ghana [33,58], South Africa [60], and Sub-Saharan Africa [50]. This may be explained by misconceptions, stigma, low education levels, and cultural beliefs that reduce recognition of symptoms and delay care-seeking. These findings highlight the need for strengthened public Health education and awareness campaigns to promote early detection and timely Healthcare utilization. However, the evidence is based on a limited number of studies, although consistency across settings supports this association.

The reviewed studies showed that the absence of pain was significantly associated with delayed Health-seeking behavior among cancer patients [8,28], which is similar to studies done in Malaysia [61] and the USA [62]. These findings highlight the importance of educating the public about cancer symptoms, emphasizing that the absence of pain does not necessarily indicate the absence of disease. Healthcare providers should actively engage in discussions with patients about potential cancer symptoms, regardless of pain presence, to encourage timely medical consultation and improve early detection rates.

The study found that visiting a traditional/spiritual healer was significantly associated with delayed Health-seeking behavior among cancer patients, as the patients who visited a traditional/spiritual healer were 2.63 times more likely to delay seeking Healthcare compared to those who had not visited a traditional healer [28]. The finding is similar to a study done in Sub-Saharan Africa [50], Indonesia [63], Bangladesh [57], and the USA [64]. This may be explained by cultural beliefs, trust in alternative healing practices, and limited access to formal Healthcare, which can delay timely medical consultation. These findings highlight the importance of culturally sensitive interventions, including collaboration between traditional healers and formal Healthcare systems, and community education to promote early cancer detection and timely care. However, the evidence is based on a limited number of studies, although consistent findings support the relevance of this factor.

### Implications

Evidence from other sub-Saharan African countries demonstrates that culturally sensitive, community-based cancer control strategies are both feasible and effective. Experiences from Ghana, Uganda, Rwanda, Kenya, and South Africa [65–68] highlight the successful integration of community actors, such as traditional healers and community Health workers, into formal Healthcare systems to promote early cancer detection and timely care-seeking. From a theoretical perspective, these findings align with Health behavior and access frameworks, which emphasize the role of sociocultural context, community support, and system-level accessibility in shaping Health-seeking behavior. Clinically, adapting such models to the Ethiopian context could strengthen early diagnosis, improve patient navigation through the Healthcare system, and reduce delays in care. Therefore, integrating culturally appropriate interventions within primary Healthcare, enhancing community engagement, and strengthening referral systems may significantly improve cancer outcomes in Ethiopia.

## Strength and Limitation

This systematic review and meta-analysis provide a comprehensive synthesis of delayed Health-seeking behavior and its associated factors among cancer patients in Ethiopia. Key strengths include the inclusion of studies from both hospital and community settings, adherence to PRISMA 2020 guidelines, PROSPERO registration, and the use of standardized quality appraisal tools and appropriate statistical methods, which enhance the reliability of the findings.

However, several limitations should be considered. First, all included studies were cross-sectional, limiting the ability to establish causal relationships. Second, there was variability in the operational definition of delayed Health-seeking behavior across studies, which may have introduced heterogeneity. Third, the small number of included studies (n = 7) reduces the statistical power of publication bias assessments and may affect the precision of pooled estimates. Additionally, most studies were conducted in specific regions and healthcare settings, which may limit the generalizability of the findings to the entire country. A further limitation is the imbalance between adult and pediatric evidence, as only one study focused on pediatric patients. Although sensitivity analysis showed that excluding this study did not substantially change the pooled estimate, the lack of pediatric data limits age-specific interpretation. Future research should include more pediatric-focused studies to better understand delays across different age groups.

## Conclusion and recommendation

This systematic review and meta-analysis revealed that half of cancer patients in Ethiopia experienced delayed health-seeking behavior. Delayed care-seeking was associated with older age, rural residence, low income, lack of health insurance, poor knowledge and awareness of cancer, low social support, absence of pain symptoms, and visiting traditional or spiritual healers. These findings indicate that both structural barriers and sociocultural factors contribute to delayed care-seeking. Based on these findings, interventions should prioritize improving cancer awareness and knowledge, particularly at the community level; expanding access to affordable Healthcare services, including strengthening Health insurance coverage; and enhancing rural Healthcare infrastructure and referral systems. In addition, integrating psychosocial support into cancer care and promoting collaboration with traditional and community actors may help address sociocultural barriers identified in this review. Targeted strategies addressing these determinants are essential to reduce delays and improve timely cancer diagnosis in Ethiopia. Given the limited evidence from pediatric populations, further studies are needed to better understand Health-seeking delays among children with cancer in Ethiopia.

### Highlights of the Studies

What is already known

- Delayed Health-seeking behavior among cancer patients leads to late diagnosis, poorer prognosis, and higher mortality, particularly in low- and middle-income countries like Ethiopia.
- Socio-demographic (age, residence), economic (income, insurance), Healthcare access, and cultural factors (reliance on traditional healers) influence delays in care-seeking.
- Previous studies in Ethiopia provided fragmented evidence, mostly region-specific, without a consolidated national-level synthesis.

What this paper adds to the existing body of knowledge

- This paper provides the first national-level systematic review and meta-analysis on delayed Health-seeking behavior among cancer patients in Ethiopia, with a pooled prevalence of 52%.
- It identifies key associated factors (e.g., rural residence, low income, poor knowledge, lack of insurance, visiting traditional healers) influencing delays in seeking cancer care.
- It fills a major evidence gap by consolidating fragmented regional studies and offers targeted public Health recommendations to improve early cancer detection and outcomes.

## Supporting information

S1 File PRISMA checklist for the systematic review and meta-analysis on Delayed Health-Seeking Behavior and Its Associated Factors among Cancer Patients in Ethiopia, 2025. (DOCX)
