# Supplementary material for: Delayed health-seeking behavior and its associated factors among cancer patients in Ethiopia: A systematic review and meta-analysis, 2025
Source: PLoS One. 2026 Jul 2;21(7):e0352869. doi: 10.1371/journal.pone.0352869 (PMC13327241; doi:10.1371/journal.pone.0352869)
Supplement: S1 File — (DOCX) [file pone.0352869.s001.docx]

| **Section and Topic** | **Item #** | **Checklist item** | **Location where item is reported** |
| --- | --- | --- | --- |
| **TITLE** | | |  |
| Title | 1 | The title clearly identifies the report as a systematic review and meta-analysis focusing on delayed health-seeking behavior and its associated factors among cancer patients in Ethiopia. | Page 1 |
| **ABSTRACT** | | |  |
| Abstract | 2 | A structured abstract is provided, including background, objectives, data sources, eligibility criteria, methods of synthesis, main results (pooled prevalence and pooled adjusted odds ratios), and conclusions. | Page 2 |
| **INTRODUCTION** | | |  |
| Rationale | 3 | The introduction describes delayed health-seeking behavior as a major contributor to late cancer diagnosis and poor outcomes in low- and middle-income countries, highlighting the lack of pooled national evidence in Ethiopia that justifies this review. | Page 3 |
| Objectives | 4 | The objective is clearly stated as estimating the pooled prevalence of delayed health-seeking behavior and identifying its associated factors among cancer patients in Ethiopia. | Page 4 |
| **METHODS** | | |  |
| Eligibility criteria | 5 | Observational studies conducted in Ethiopia that reported delayed health-seeking behavior and/or its associated factors among cancer patients were included. Reviews, editorials, case reports, qualitative-only studies, and non-human studies were excluded. | Page 5 |
| Information sources | 6 | A comprehensive search was conducted in PubMed, Scopus, Web of Science, Google Scholar, African Journals Online, and Ethiopian institutional repositories. | Page 6 |
| Search strategy | 7 | A detailed search strategy using combinations of controlled vocabulary and free-text terms related to cancer, delayed health-seeking behavior, diagnostic delay, and Ethiopia, combined with Boolean operators, was applied. | Page 7 |
| Selection process | 8 | Two reviewers independently screened titles, abstracts, and full-text articles for eligibility. Discrepancies were resolved through discussion and consensus. | Page 8 |
| Data collection process | 9 | Data were extracted independently by two reviewers using a standardized and pretested data extraction form | Page 9 |
| Data items | 10a | The primary outcomes were the prevalence of delayed health-seeking behavior and adjusted odds ratios for factors associated with delay. All compatible effect estimates reported in each study were collected. | Page 10 |
|  | 10b | Additional variables extracted included author, year, region, study design, sample size, cancer type, operational definition of delay, and study population characteristics. When information was unclear, authors’ definitions were followed without imputation. | Page 10 |
| Study risk of bias assessment | 11 | Risk of bias was assessed using the Joanna Briggs Institute (JBI) Critical Appraisal Checklist by two independent reviewers. Disagreements were resolved by consensus. | Page 11 |
| Effect measures | 12 | Pooled prevalence estimates and pooled adjusted odds ratios (AORs) with 95% confidence intervals were used as effect measures. | Page 12 |
| Synthesis methods | 13a | Studies were grouped for synthesis based on similarity in outcomes and study design. Only studies reporting comparable measures were included in each meta-analysis. | Page 13 |
|  | 13b | Where necessary, effect estimates were log-transformed and standard errors calculated prior to meta-analysis. | Page 13 |
|  | 13c | Forest plots and summary tables were used to visually present individual study results and pooled estimates. | Page 13 |
|  | 13d | A random-effects meta-analysis model was applied. Statistical heterogeneity was assessed using the I² statistic, and analyses were conducted using R software. | Page 14 |
|  | 13e | Subgroup analyses were conducted based on cancer type, age group, and study region to explore potential sources of heterogeneity. | Page 14 |
|  | 13f | Sensitivity analyses were performed by excluding individual studies to assess the robustness of the pooled estimates. | Page 14 |
| Reporting bias assessment | 14 | Publication bias was assessed using funnel plot symmetry and Egger’s regression test | Page 15 |
| Certainty assessment | 15 | Formal certainty of evidence assessment (e.g., GRADE) was not conducted, and this is acknowledged as a limitation. | Page 15 |
| **RESULTS** | | |  |
| Study selection | 16a | The study selection process is summarized using a PRISMA flow diagram, detailing the number of records identified, screened, excluded, and included. | Page 16; Figure 1 |
|  | 16b | Studies excluded after full-text review are described with reasons for exclusion. | Page 16 |
| Study characteristics | 17 | Characteristics of all included studies, including author, year, region, design, sample size, cancer type, definition of delay, and prevalence, are presented in a table. | Page 17; Table 1 |
| Risk of bias in studies | 18 | Results of the JBI risk of bias assessment for each included study are presented. | Page 18; Table 2 |
| Results of individual studies | 19 | For each study, prevalence estimates and adjusted odds ratios with 95% confidence intervals are presented using tables and forest plots. | Page 19 |
| Results of syntheses | 20a | The characteristics and methodological quality of studies contributing to each synthesis are summarized. | Page 20 |
|  | 20b | Pooled prevalence and pooled adjusted odds ratios are reported with corresponding 95% confidence intervals and heterogeneity statistics. | Page 20 |
|  | 20c | Results of subgroup analyses exploring heterogeneity by cancer type, age group, and region are presented. | Page 21 |
|  | 20d | Sensitivity analysis results demonstrate the stability of pooled estimates. | Page 21 |
| Reporting biases | 21 | Funnel plots and Egger’s test results assessing reporting bias are presented. | Page 21 |
| Certainty of evidence | 22 | Certainty of evidence was not formally assessed and is acknowledged as a limitation of the review. | Page 22 |
| **DISCUSSION** | | |  |
| Discussion | 23a | The findings are interpreted in relation to existing regional and global evidence on delayed cancer care. | Page 23 |
|  | 23b | Limitations of the included studies, including heterogeneity and variability in delay definitions, are discussed. | Page 24 |
|  | 23c | Limitations of the review process, including the small number of studies, are acknowledged. | Page 24 |
|  | 23d | Implications for clinical practice, health policy, and future research are discussed. | Page 25 |
| **OTHER INFORMATION** | | |  |
| Registration and protocol | 24a | The review protocol was registered in PROSPERO and the registration number is reported. | Page 5 |
|  | 24b | The PROSPERO registry is cited as the location of the accessible protocol. | Page 5 |
|  | 24c | No protocol amendments were made after registration. | Page 5 |
| Support | 25 | Sources of financial and non-financial support and the role of funders are described. | Page 26 |
| Competing interests | 26 | All authors declare no competing interests. | Page 26 |
| Availability of data, code and other materials | 27 | Data extracted from included studies, analytic datasets, and supplementary materials are made available within the manuscript and supporting information files. | Page 26 |

*From:*  Page MJ, McKenzie JE, Bossuyt PM, Boutron I, Hoffmann TC, Mulrow CD, et al. The PRISMA 2020 statement: an updated guideline for reporting systematic reviews. BMJ 2021;372:n71. doi: 10.1136/bmj.n71. This work is licensed under CC BY 4.0. To view a copy of this license, visit <https://creativecommons.org/licenses/by/4.0/>
