# Supplementary material for: Delayed health-seeking behavior and its associated factors among cancer patients in Ethiopia: A systematic review and meta-analysis, 2025
Source: PLoS One. 2026 Jul 2;21(7):e0352869. doi: 10.1371/journal.pone.0352869 (PMC13327241; doi:10.1371/journal.pone.0352869)
Supplement: Attachment — Submitted filename: Response to Reviewers.docx [file pone.0352869.s005.docx]

# Response to Academic Editor

Manuscript ID: PONE-D-25-30632R2

Title: Delayed Health-Seeking Behavior and Its Associated Factors among Cancer Patients in Ethiopia: A Systematic Review and Meta-Analysis, 2025

Dear Academic Editor,

We sincerely thank you for your careful evaluation of our revised manuscript and for your valuable comments. We are grateful for the positive assessment from both reviewers and have carefully addressed all remaining editorial concerns.

**Comment 1: Conclusion**

**Editor Comment:** “In conclusion rather than stating ‘Delayed Health-seeking behavior was common among Ethiopian cancer patients, with a pooled prevalence of 52%’ state it as ‘Our review showed that half of cancer patients …………….’”

**Author Response:** Thank you for this helpful suggestion. We have revised the conclusion accordingly to improve clarity and readability. Revision made: “Our review showed that approximately half of cancer patients in Ethiopia experienced delayed health-seeking behavior. Delayed care-seeking was associated with rural residence, poor knowledge, limited awareness of cancer, and absence of pain symptoms.”

**Comment 2: Results/Figures**

**Editor Comment:** “Once again, critically review all figures for accuracy and clarity. The current presentation still falls below the expected standard. For example, in Figure 2, the list of authors is presented without an appropriate subheading such as ‘Authors’ above the names. Similarly, the proportions and their corresponding 95% confidence intervals are listed without a clear subtitle or heading indicating ‘Proportions with 95% CI.’”

**Author Response:** Thank you for identifying this issue. We carefully reviewed all figures and revised them to improve internal labeling, structure, readability, and consistency. We reviewed and standardized all figure labels, headings, fonts, spacing, alignment, and axis descriptions. These revisions have substantially improved the clarity and presentation quality of all figures. We appreciate the editor’s careful review and believe the manuscript has been strengthened as a result of these revisions.

**Response to Reviewers**

**Response to Reviewer #1**

We sincerely thank the reviewer for carefully re-evaluating our manuscript and for acknowledging that all previous comments have been adequately addressed. We greatly appreciate your constructive feedback throughout the review process, which significantly improved the quality of our manuscript.

**Response to Reviewer #2**

We are grateful for the reviewer’s positive assessment of our revised manuscript. Thank you for recognizing the improvements in methodological transparency, structure, and writing quality. Your valuable comments have strengthened the manuscript, and we appreciate your support and encouraging remarks.
