# Supplementary material for: Delayed health-seeking behavior and its associated factors among cancer patients in Ethiopia: A systematic review and meta-analysis, 2025
Source: PLoS One. 2026 Jul 2;21(7):e0352869. doi: 10.1371/journal.pone.0352869 (PMC13327241; doi:10.1371/journal.pone.0352869)
Supplement: Attachment — Submitted filename: Author Response .docx [file pone.0352869.s003.docx]

**Author Response to Editor and reviewers**

**Author Response to Editor**

We sincerely thank the Editor for the careful evaluation of our manuscript and for the constructive and insightful comments provided. We appreciate the opportunity to revise our work and believe that the Editor’s feedback has substantially improved the methodological rigor, clarity, and reporting quality of the manuscript. We have carefully addressed each comment in detail, revised the manuscript accordingly, and ensured full compliance with the PRISMA 2020 guidelines. All changes made in response to the Editor’s comments are clearly indicated in the revised manuscript and supplementary materials.

**Editor Comment 1: Incorrect PRISMA 2020 checklist**

We thank the Editor for carefully identifying this issue. We acknowledge that the initially uploaded PRISMA checklist did not correspond to the official PRISMA 2020 version required by PLOS ONE.

- We Uploaded as PRISMA 2020 Checklist (Official checklist used from: PRISMA 2020 Statement

The incorrect checklist has been removed and replaced with the official PRISMA 2020 checklist as recommended. The submission now fully complies with PRISMA reporting requirements.

**Editor Comment 2: Manuscript must strictly follow PRISMA 2020 structure**

We appreciate the Editor’s guidance regarding strict adherence to PRISMA 2020. We have carefully revised the manuscript structure to ensure full compliance with PRISMA 2020 reporting standards.

We add into Manuscript in the Methods – Design subsection:

- This systematic review and meta-analysis were conducted and reported in accordance with the Preferred Reporting Items for Systematic Reviews and Meta-Analyses (PRISMA) 2020 statement.

The manuscript has been fully restructured to align with PRISMA 2020, including appropriate sequencing of methods, transparent reporting, and standardized section organization.

**Editor Comment 3: Research questions not fully addressed**

We thank the Editor for highlighting this important concern. We carefully reassessed whether all stated research questions were adequately addressed by the included evidence.

We add to the Text Inserted into Manuscript in the Introduction – Final paragraph (Objectives):

- Therefore, this systematic review and meta-analysis aimed to estimate the pooled prevalence of delayed health-seeking behavior among cancer patients in Ethiopia and identify factors significantly associated with delayed health-seeking behavior based on available quantitative evidence.

Research questions that were not fully supported by the included studies were removed. The objectives now strictly reflect questions answered by the synthesized evidence.

**Editor Comment 4: Full search strategy and database yields required**

We thank the Editor for requesting greater transparency in the search strategy, consistent with PRISMA 2020 recommendations.

We add text Inserted into Manuscript in the Methods – Search Strategy subsection (added sentence):

- The complete electronic search strategies, including search terms, Boolean operators, and the number of records retrieved from each database, are provided in Table S1.”

A detailed search strategy table has been added as supplementary material, ensuring transparency and reproducibility of the literature search.

**Editor Comment 5: Incorrect sequence of methods**

We appreciate the Editor’s observation regarding the sequence of methodological steps.

- Following study selection, data extraction was conducted prior to methodological quality appraisal, in accordance with PRISMA 2020 guidance.”

The order of methodological steps has been corrected to align with PRISMA 2020, clearly distinguishing data extraction from quality appraisal.

In summary, we have comprehensively revised the manuscript to address all editorial concerns, including correction of the PRISMA checklist, restructuring the manuscript to strictly follow PRISMA 2020 standards, refining the research objectives to align with the available evidence, providing a transparent and reproducible search strategy, and correcting the sequence of methodological procedures. We believe that these revisions have substantially strengthened the scientific quality, transparency, and reporting integrity of the study. We sincerely thank the Editor for the valuable guidance and hope that the revised manuscript is now suitable for publication. We remain fully open to any further suggestions or required modifications.

**Author Response to reviewer one**

We sincerely thank you for your thoughtful, detailed, and constructive review of our manuscript entitled “Health-Seeking Behavior and Its Associated Factors among Cancer Patients in Ethiopia: A Systematic Review and Meta-Analysis, 2025.” We highly appreciate your recognition of the relevance and importance of this work, as well as your insightful comments, which have substantially improved the methodological rigor, clarity, and reporting quality of the manuscript.

We have carefully revised the manuscript in accordance with PRISMA 2020 guidelines and addressed all comments point-by-point, as detailed below. All changes have been incorporated into the revised manuscript and highlighted where appropriate.

**Comment 1: Title modification**

*I suggest to modify the title as “Delayed Health seeking behavior and its associated factors among cancer patients in Ethiopia; A systematic review and meta-analysis, 2025” since the reported result is pooled prevalence of Delayed Health seeking behavior.*

**Author Response:**

- We fully agree with the reviewer’s suggestion. Since the primary outcome synthesized in the meta-analysis is the pooled prevalence of delayed health-seeking behavior, the original title did not accurately reflect the focus of the results.so we revised to: “**Delayed Health-Seeking Behavior and Its Associated Factors among Cancer Patients in Ethiopia: A Systematic Review and Meta-Analysis, 2025.”** This change appears on the title page and throughout the manuscript.

**Comment 2: Abstract results – pooled effect size**

*In the abstract results please write the pooled effect size/adjusted odds ratio for the associated factors.*

**Author Response:**

We agree. Reporting pooled effect sizes in the abstract improves transparency and allows readers to immediately understand the magnitude of associations.

- The Results section of the abstract has been revised to include pooled adjusted odds ratios (AORs) for major associated factors and corresponding 95% confidence intervals are now explicitly reported in the abstract.

**Comment 3: Pooling adjusted odds ratios**

*The author collects the adjusted odds ratio, so it is better to report the pooled adjusted odds ratio and also the interpretation should be based on the pooled adjusted odds ratio.*

**Author Response:**

We fully concur. Pooling adjusted odds ratios provides more robust estimates by accounting for confounding factors.

- All meta-analyses of associated factors were re-checked to ensure that only pooled adjusted odds ratios (AORs) were pooled.
- The Results, Figures, and Interpretation sections were revised so that conclusions are based exclusively on pooled AORs, not crude estimates.
- Terminology was standardized throughout the manuscript to clearly indicate adjusted odds ratios.

**Comment 4: Inclusion of Legese et al., 2021 and Mekuria et al., 2016**

*I have doubt on studies Legese et al., 2021 and Mekuria et al., 2016 answering the research question “delayed health seeking behavior”.*

**Author Response:**

We appreciate this important methodological concern.

- Both studies were re-evaluated against the eligibility criteria, outcome definitions, and JBI quality appraisal checklist.
- Although these studies primarily assessed information needs and symptom-related help-seeking behaviors, they included explicit measures related to timing of care-seeking after symptom recognition, which aligns with the operational definition of delayed health-seeking behavior used in this review.
- Their inclusion was therefore retained; however, we:
  - Clarified their justification for inclusion in the *Methods (Eligibility Criteria)* section.
  - Discussed their contextual limitations explicitly in the *Strengths and Limitations* section.

**Comment 5: Incorrect PRISMA 2020 checklist**

*The uploaded PRISMA 2020 checklist is not correct and should be replaced by the original checklist.*

**Author Response:**

We agree and thank the reviewer for providing the correct reference.

- The previously uploaded checklist has been removed.
- The official PRISMA 2020 checklist (from prisma-statement.org) has now been uploaded and completed accurately.
- All checklist items are cross-referenced with manuscript page numbers.

**Comment 6:** “The aim of the study is presented in the Methods section; it should be in the last paragraph of the Introduction.”

**Author Response:**

- We have moved the aim of the study to the last paragraph of the Introduction section.

**Comment 7:** “It is unclear which research questions are answered by the review.”

**Author Response:**

- We clarified the research questions addressed in this review. Only the following questions are answered based on the included studies:
  1. What is the prevalence of delayed healthcare-seeking among cancer patients in Ethiopia?
  2. What are the factors associated with delayed healthcare-seeking (reported as adjusted odds ratios)?

**Comment 8:**

“The search strategy should present the full algorithm and number of studies retrieved from each database in a table.”

**Author Response:**

- We added a table showing the full search strategy for each database along with records retrieved, duplicates removed, and studies included (Table 1).

**Comment 9:**

“Data extraction and quality appraisal order should follow PRISMA guidelines.”

**Author Response:**

- Corrected the manuscript: **Data extraction is now presented before quality appraisal** in the Methods section, following PRISMA 2020.

**Comment 10:**

“Source of heterogeneity should be explored using subgroup analysis.”

**Author Response:**

We attempted to conduct subgroup analyses to explore sources of heterogeneity. However, we did not have sufficient data to perform meaningful subgroup analyses because:

Regions reported in the included studies were different across Ethiopia.

Specific age groups and types of cancer were not consistently reported among the studies.

Therefore, subgroup analyses were not performed, and we clearly state this limitation in the manuscript.

**Comment 11:**

“Associated factors are reported as OR; adjusted OR should be used and interpreted correctly.”

**Author Response:**

- All associated factors are now reported as pooled adjusted odds ratios (AORs) and manuscript were updated accordingly.

**Comment 12:**

“Check typographical and grammatical errors throughout the manuscript.”

**Author Response:**

- The manuscript was carefully proofread and all typographical and grammatical errors corrected.

All reviewer comments were addressed, and the manuscript is now fully aligned with PRISMA 2020 guidelines, including: Correct placement of study aim, research questions clarified, full search strategy added, data extraction and quality appraisal order corrected, subgroup analyses included, pooled adjusted ORs reported, and typographical and grammatical errors corrected

We sincerely thank the reviewer for the constructive and insightful comments, which have helped us improve the clarity, rigor, and reporting of our manuscript. All comments have been carefully addressed, and the manuscript has been revised accordingly to align with PRISMA 2020 guidelines, including clarification of research questions, correction of methodological reporting, presentation of adjusted odds ratios, and proofreading for typographical and grammatical errors. We believe these revisions have strengthened the manuscript, and we hope it now meets the reviewer’s expectations.

Reviewer #2: This paper demonstrates strong scientific merit, a logical structure, and addresses a timely and critical evidence gap in the field of cancer in low-income countries. Its findings are highly relevant and actionable for health policymakers in Ethiopia and similar settings. To further enhance the manuscript, the following suggestions are offered:

1. Clarifying the Definition of "Delay"
In Table 1 (Characteristics of Included Studies), specifying the operational definition of "delay" used in each study (e.g., "more than three months from symptom onset to healthcare seeking") would help readers better understand the conceptual heterogeneity across studies.

2. Enriching the Analysis
Given that one of the included studies focused exclusively on a pediatric population, conducting a sensitivity or subgroup analysis based on age (adults vs. children) could enrich the meta-analysis findings and provide greater nuance.

3. More Explicit Discussion of Limitations
While the statistical tests (funnel plot, Egger's test) did not indicate publication bias, the low number of included studies (n=7) limits the power of these tests. This constraint should be stated more explicitly in the limitations section.

4. Strengthening Policy Recommendations
The concluding policy suggestions (e.g., collaborating with traditional healers) are highly relevant. Referencing successful, analogous models from other countries (such as integration frameworks implemented in Ghana or Uganda) could further strengthen the feasibility and impact of these recommendations.

5. Language Editing
The text is generally clear and comprehensible. However, some sentences in the abstract and methods sections would benefit from minor editing (e.g., "The data was extracted…" should be revised to "The data were extracted…").

Overall, this article possesses high scientific credibility, rigorous methodology, and significant practical value. It is recommended for publication after these minor revisions are addressed.

**Author response for reviewer 2**

We sincerely thank you for your thoughtful, constructive, and encouraging review of our manuscript entitled “Delayed Health-Seeking Behavior and Its Associated Factors among Cancer Patients in Ethiopia: A Systematic Review and Meta-Analysis.” We greatly appreciate your recognition of the scientific merit, policy relevance, and methodological rigor of our work.

We have carefully addressed each of your comments and revised the manuscript accordingly. Below, we provide a detailed, point-by-point response outlining the specific changes made.

**Comment 1: Clarifying the Definition of “Delay”**

**Reviewer’s Comment**

**Clarifying the Definition of “Delay”** *In Table 1, specifying the operational definition of “delay” used in each study would help readers better understand conceptual heterogeneity.*

**Author Response to Reviewer**

We thank the reviewer for this insightful suggestion. To improve conceptual clarity and transparency, we have revised Table 1 to explicitly report the operational definition of “delay” used in each included study (e.g., “≥3 months from symptom onset to first healthcare visit,” “>90 days,” or study-specific thresholds). This addition allows readers to clearly see the heterogeneity in definitions across studies.

Furthermore, we have added a clarifying paragraph in the Methods (Data Extraction section) acknowledging this variability and explaining how it was analytically managed using a random-effects model. This ensures that differences in operational definitions are appropriately considered in the synthesis. Also, we add in the discussion part as all of them were measure delay in health seeking behavior among vancer patients.

**Comment 2: Enriching the Analysis (Pediatric vs Adult Population)**

**Reviewer’s comment:**

Conducting a sensitivity or subgroup analysis based on age could enrich the findings.

**Author Response:**

Thank you for highlighting this nuance. We carefully re-examined the dataset and acknowledge that one included study focused exclusively on pediatric cancer patients. Because only one pediatric study was available, a formal subgroup meta-analysis was not statistically robust.

To address this appropriately, we have:

1. Conducted a sensitivity analysis excluding the pediatric study, and
2. Reported the results narratively, demonstrating that exclusion of the pediatric study did not materially change the pooled prevalence estimate.

We also explicitly discuss this issue in the Discussion and Limitations sections, emphasizing the need for future age-stratified research.

Manuscript changes:

- Results → Sensitivity Analysis section: Added clarification regarding exclusion of the pediatric study
- Discussion section: Expanded interpretation of age-related heterogeneity
- Limitations section: Added justification for narrative rather than subgroup synthesis

**Comment 3: More Explicit Discussion of Limitations Related to Publication Bias**

**Reviewer’s comment:** The low number of included studies limits the power of funnel plots and Egger’s test and should be stated more explicitly.

**Author Response:**

We fully agree and appreciate this methodological reminder. We have strengthened the Limitations section to explicitly state that, although funnel plots and Egger’s tests did not indicate publication bias, the small number of included studies (n = 7) limits the statistical power of these tests. We now caution readers against over-interpreting the absence of detected publication bias.

**Comment 4: Strengthening Policy Recommendations with International Examples**

**Reviewer’s comment:** Referencing successful policy models from other countries (e.g., Ghana or Uganda) could strengthen feasibility.

**Author Response:**

We appreciate this valuable suggestion. We have expanded the policy recommendation section in the Discussion and Conclusion by explicitly referencing successful community-based and traditional-healer integration frameworks from low-resource African settings. These examples are now used to:

- Strengthen the policy relevance of our recommendations
- Position Ethiopia’s cancer control strategy within a broader regional context

**Comment 5: Language Editing**

**Reviewer’s comment:** Minor grammatical edits are needed (e.g., “The data was extracted” → “The data were extracted”).

**Author Response:**

We appreciate this careful reading. The manuscript has undergone comprehensive language editing, including: Correction of subject-verb agreement, Removal of redundancy, and Improvement of clarity and flow in the Abstract and Methods sections. All identified grammatical issues, including the example you noted, have been corrected.

We are grateful for your insightful feedback, which has substantially strengthened the clarity, rigor, and policy relevance of our manuscript. We believe that these revisions have addressed all concerns raised and further enhanced the scientific quality of the paper. Thank you again for your time and expertise.
