# Supplementary material for: Delayed health-seeking behavior and its associated factors among cancer patients in Ethiopia: A systematic review and meta-analysis, 2025
Source: PLoS One. 2026 Jul 2;21(7):e0352869. doi: 10.1371/journal.pone.0352869 (PMC13327241; doi:10.1371/journal.pone.0352869)
Supplement: Attachment — Submitted filename: Response to the reviewers.docx [file pone.0352869.s004.docx]

**RESPONSE TO EDITOR AND REVIEWER 2 (PLOS ONE)**

**RESPONSE TO EDITOR**

we sincerely thank the editor and reviewer for their constructive and insightful comments. We have carefully revised the manuscript to improve clarity, methodological transparency, and scientific rigor. All comments have been addressed point-by-point below, and corresponding changes have been incorporated into the revised manuscript with tracked changes.

**Abstract comments**

**Comment 1: mention databases in methods**

**Author response:**
thank you for this important suggestion. We have revised the methods section of the abstract to explicitly include the databases searched. And we included as “a systematic search was conducted in PubMed, Scopus, web of science, google scholar, African journals online (AJOL), and Ethiopian university repositories.”

**Comment 2: include total participants**

**Author response:** we agree and have added the total pooled sample size and we reinsert to the result section as “a total of 2,641 participants from seven studies were included.”

**Comment 3: “conclusion” → “conclusions”**

**Author response:** we corrected **conclusion” → “conclusions**

**Comment 4: justify “highly prevalent”**

**Author response:** we appreciate this concern. We replaced subjective wording with evidence-based interpretation and we stated as “delayed health-seeking behavior was common among ethiopian cancer patients, with a pooled prevalence of 52%.”

**Comment 5: conclusion not aligned with findings**

**Author response:** we revised the conclusion to strictly reflect results. We revised abstract **conclusion as** “delayed health-seeking behavior is common among cancer patients in Ethiopia (52%) and is significantly associated with rural residence, poor knowledge, lack of awareness, and absence of symptoms such as pain.”

**Comment 6: what does “quantify” mean?**

**Author response:** we clarified wording as we assumed before the quantify word means was it estimates the magnitude of key associated factors using pooled effect sizes (odds ratios).”, but we remove the word quantify from the manuscript under highlight.

**Introduction comments**

**Comment: poor paragraph structure**

**Author response:** we restructured the introduction into coherent paragraphs (no single-sentence paragraphs).

**Comment: poor logical flow Health-seeking behaviors – global studies. Https://fiveable.me/hs-global-studies/key-terms/health-seeking-behaviors**

**Author response:** we reorganized from: global → LMIC → sub-Saharan Africa → Ethiopia → gap

**Comment: add who / UNICEF / Ethiopia initiatives**

**Author response:** added new paragraph as “at the policy level, global and national initiatives have emphasized early detection and timely care-seeking as key strategies for reducing cancer mortality (20). The world health organization (who) launched the global cancer initiative to promote early detection, timely diagnosis, and effective treatment as core pillars for reducing cancer-related deaths (21–23). In Ethiopia, the national cancer control plan prioritizes early diagnosis, expansion of oncology services, and public awareness campaigns to address delays in care (24). However, despite these initiatives, significant gaps remain in implementation, particularly in addressing behavioral, cultural, and socioeconomic barriers influencing health-seeking behavior (9,10,19).”

**Comment: clearly state research gap**

**Author response:** we strengthened the gap and we replace “existing studies in Ethiopia suggest that delayed health-seeking behavior is influenced by a complex interplay of socio-demographic, economic, cultural, and health system factors (11,15–17). However, the available evidence is fragmented, region-specific, and methodologically heterogeneous, limiting its applicability for national-level decision-making. Moreover, inconsistencies in definitions and measurements of delay further complicate the interpretation of findings across studies.”

**Methods comments**

**Comment: why no gray literature?**

**Author** **response:** we acknowledge the importance of gray literature in minimizing publication bias. In our review, we attempted to identify gray literature; however, no eligible studies meeting our inclusion criteria were identified. Additionally, we exercised caution in including non–peer-reviewed sources due to concerns about methodological rigor and potential bias. Therefore, gray literature was not included in the final analysis. This has now been clarified in the methods section.

**Comment: how were repositories searched?**

**Author response:** institutional repositories were searched manually using predefined keywords, and relevant theses and dissertations were screened based on eligibility criteria.”

**Comment: keywords vs mesh**

**Author response:** we clarified. “Mesh terms were used in PubMed to ensure standardized indexing, while equivalent keywords were applied across other databases to maximize sensitivity.” Our search strategy combined both free-text keywords and medical subject headings (mesh) terms to maximize sensitivity and specificity. Keywords were used to capture recently published or non-indexed articles, while mesh terms ensured retrieval of articles indexed under standardized subject headings. Boolean operators (“and,” “or”) were applied to appropriately combine these terms and ensure a comprehensive search.

**Results comments**

**Comment: PRISMA inconsistency**
**author response:**
we sincerely thank the reviewer for highlighting this issue. We carefully verified and corrected all numerical inconsistencies to ensure alignment across the text, table 1, and the PRISMA diagram.

**Comment: figures unclear**
**author response:**
we thank the reviewer for this important observation. All figures and their captions have been thoroughly revised to improve clarity, completeness, and consistency to line with journal requirements.

**Discussion comments**

**Comment: lacks depth**
**author response:**
we appreciate this valuable suggestion. The discussion has been substantially expanded by incorporating deeper explanations related to health system factors, sociocultural context, and access barriers, as well as relevant theoretical perspectives to strengthen interpretation of the findings.

**Comment: explain discrepancies**
**author response:**
we thank the reviewer for this insightful comment. We have added explanations for observed discrepancies by considering differences in health system capacity, socioeconomic conditions, and cultural contexts across settings.

**Comment: add implications**
**author response:**
we are grateful for this suggestion. The implications section has been strengthened to clearly outline both clinical and policy implications derived from the study findings.

**Comment: strength & limitation**
**author response:**
we thank the reviewer for this comment. The strengths and limitations section has been refined to improve clarity, structure, and completeness

**Conclusion comments**

**Comment: avoid general statements**
**author response:**
we appreciate this guidance. The conclusion has been revised to ensure it is strictly based on the findings of the current study.

**Comment: recommendations must align**
**author response:**
we thank the reviewer for this important point. The recommendations have been revised to align directly with the identified factors, including rural access, knowledge, awareness, and financial barriers.

**General comments**

**Comment: English editing**
**author response:**
we sincerely thank the reviewer for this suggestion. The manuscript has undergone professional language editing to improve clarity and readability.

**Comment: old references**
**author response:**
we appreciate this comment. References have been updated where possible, and older seminal works have been retained only when necessary.

**Comment: add line numbers**
**author response:**
we thank the reviewer for this note. Line numbers have been added to the revised manuscript as requested.

We believe that the revisions made in response to the reviewers’ comments have substantially strengthened the manuscript. We hope that the revised version meets the journal’s standards and expectations. We sincerely appreciate the time and effort invested by the editor and reviewers in evaluating our work, and we look forward to your favorable consideration.

**AUTHOR RESPONSES TO REVIEWER #2**

We sincerely thank Reviewer #2 for the thorough evaluation of our manuscript and for the constructive and insightful comments. We greatly appreciate the positive feedback regarding the relevance of the topic and the usefulness of our findings. We have carefully addressed each comment and revised the manuscript accordingly to improve clarity, methodological transparency, and overall quality. Our detailed responses are provided below.

**Comment 1: Operational definition of delay**

**Reviewer Comment:** The Methods section lacks a clear summary of how delay was defined and handled across studies.

**Author Response:** We thank the reviewer for this important observation. We have now added a clear summary in the Data Extraction subsection describing the variation in operational definitions of delay across studies and how this heterogeneity was addressed analytically.

**Comment 2: Search strategy dates**

**Reviewer Comment:** Search dates are missing.

**Author Response:** We sincerely thank the reviewer for highlighting this important reporting requirement. The search dates have now been clearly specified in Table 1 as a footnote. All database searches were conducted between March 1 and March 10, 2025.

**Comment 3: Figure labels**

**Reviewer Comment:** Figures 5, 7, and 9 incorrectly use “Odds Ratio” instead of “Adjusted Odds Ratio (AOR)”.

**Author Response:** We thank the reviewer for this valuable comment. The x-axis labels in Figures 5, 7, and 9 have been revised from “Odds Ratio” to “Adjusted Odds Ratio (AOR)” to ensure consistency with the reported effect measures in the text and abstract.

**Revision made in manuscript:**

- Updated figure images:
  - Figure 5 → “Adjusted Odds Ratio (AOR)”
  - Figure 7 → “Adjusted Odds Ratio (AOR)”
  - Figure 9 → “Adjusted Odds Ratio (AOR)”

**Comment 4: Pediatric population**

**Reviewer Comment:** Add a sentence in the conclusion calling for pediatric-specific research.

**Author Response:** We appreciate this insightful suggestion. A statement has been added to the conclusion highlighting the need for further research focusing on pediatric populations.

**Comment 5: Language and grammar**

**Reviewer Comment:** Multiple grammatical errors identified.

**Author Response:** We sincerely thank the reviewer for this important comment. The manuscript has undergone thorough language revision to correct grammatical errors and improve clarity. All highlighted issues, including verb agreement and sentence structure, have been corrected throughout the manuscript.

**Examples corrected:**

- “The data was extracted” → “The data were extracted”
- “Health-seeking behavior defined as” → “Health-seeking behavior is defined as”
- “This due to” → “This is due to”

We are grateful to Reviewer #2 for the constructive feedback, which has significantly improved the quality and clarity of our manuscript. We believe that the revisions made have addressed all concerns raised and strengthened the methodological rigor and presentation of our study. We sincerely appreciate the reviewer’s time and effort and hope that the revised manuscript meets the expectations for publication.
