# Supplementary material for: Delayed health-seeking behavior and its associated factors among cancer patients in Ethiopia: A systematic review and meta-analysis, 2025
Source: PLoS One. 2026 Jul 2;21(7):e0352869. doi: 10.1371/journal.pone.0352869 (PMC13327241; doi:10.1371/journal.pone.0352869)
Supplement: Peer review history

# Peer review: Decision Letter 0

**DOI:** 10.1371/journal.pone.0352869.r001

**Authors:** Adera Debella Kebede

11 Jan 2026

PONE-D-25-30632

Health seeking behavior and its associated factors among cancer patients in Ethiopia; A systematic review and meta-analysis, 2025

PLOS One

Dear Dr. Abdulwehab,

Thank you for submitting your manuscript to PLOS ONE. After careful consideration, we feel that it has merit but does not fully meet PLOS ONE’s publication criteria as it currently stands. Therefore, we invite you to submit a revised version of the manuscript that addresses the points raised during the review process.

Please submit your revised manuscript by Feb 25 2026 11:59PM. If you will need more time than this to complete your revisions, please reply to this message or contact the journal office at plosone@plos.org. When you're ready to submit your revision, log on to https://www.editorialmanager.com/pone/ and select the 'Submissions Needing Revision' folder to locate your manuscript file.

Please include the following items when submitting your revised manuscript:

- A letter that responds to each point raised by the academic editor and reviewer(s). You should upload this letter as a separate file labeled 'Response to Reviewers'.
- A marked-up copy of your manuscript that highlights changes made to the original version. You should upload this as a separate file labeled 'Revised Manuscript with Track Changes'.
- An unmarked version of your revised paper without tracked changes. You should upload this as a separate file labeled 'Manuscript'.

If you would like to make changes to your financial disclosure, please include your updated statement in your cover letter. Guidelines for resubmitting your figure files are available below the reviewer comments at the end of this letter.

If applicable, we recommend that you deposit your laboratory protocols in protocols.io to enhance the reproducibility of your results. Protocols.io assigns your protocol its own identifier (DOI) so that it can be cited independently in the future. For instructions see: https://journals.plos.org/plosone/s/submission-guidelines#loc-laboratory-protocols. Additionally, PLOS ONE offers an option for publishing peer-reviewed Lab Protocol articles, which describe protocols hosted on protocols.io. Read more information on sharing protocols at https://plos.org/protocols?utm_medium=editorial-email&utm_source=authorletters&utm_campaign=protocols.

We look forward to receiving your revised manuscript.

Kind regards,

Adera Debella Kebede, MSC

Academic Editor

PLOS One

**Journal Requirements:**

When submitting your revision, we need you to address these additional requirements.

1. Please ensure that your manuscript meets PLOS ONE's style requirements, including those for file naming. The PLOS ONE style templates can be found at

https://journals.plos.org/plosone/s/file?id=wjVg/PLOSOne_formatting_sample_main_body.pdf and

https://journals.plos.org/plosone/s/file?id=ba62/PLOSOne_formatting_sample_title_authors_affiliations.pdf

2. We note that your Data Availability Statement is currently as follows:

“All relevant data are within the manuscript and its Supporting Information files.”

Please confirm at this time whether or not your submission contains all raw data required to replicate the results of your study. Authors must share the “minimal data set” for their submission. PLOS defines the minimal data set to consist of the data required to replicate all study findings reported in the article, as well as related metadata and methods (https://journals.plos.org/plosone/s/data-availability#loc-minimal-data-set-definition).

For example, authors should submit the following data:

- The values behind the means, standard deviations and other measures reported;

- The values used to build graphs;

- The points extracted from images for analysis.

Authors do not need to submit their entire data set if only a portion of the data was used in the reported study.

If your submission does not contain these data, please either upload them as Supporting Information files or deposit them to a stable, public repository and provide us with the relevant URLs, DOIs, or accession numbers. For a list of recommended repositories, please see https://journals.plos.org/plosone/s/recommended-repositories.

If there are ethical or legal restrictions on sharing a de-identified data set, please explain them in detail (e.g., data contain potentially sensitive information, data are owned by a third-party organization, etc.) and who has imposed them (e.g., an ethics committee). Please also provide contact information for a data access committee, ethics committee, or other institutional body to which data requests may be sent. If data are owned by a third party, please indicate how others may request data access.

3. Please include your tables as part of your main manuscript and remove the individual files. Please note that supplementary tables (should remain/ be uploaded) as separate "supporting information" files.

4. Please include captions for your Supporting Information files at the end of your manuscript, and update any in-text citations to match accordingly. Please see our Supporting Information guidelines for more information: http://journals.plos.org/plosone/s/supporting-information.

5. If the reviewer comments include a recommendation to cite specific previously published works, please review and evaluate these publications to determine whether they are relevant and should be cited. There is no requirement to cite these works unless the editor has indicated otherwise.

**Additional Editor Comments:**

The uploaded **PRISMA 2020 checklist**  is not the correct version. The author should replace it with the official PRISMA 2020 checklist available at:

https://www.prisma-statement.org/s/PRISMA_2020_checklist-k3f4.docx<svg aria-hidden="true" class="block h-[0.75em] w-[0.75em] stroke-current stroke-[0.75]" data-rtl-flip="0" height="20" width="20" xmlns="http://www.w3.org/2000/svg"><use fill="currentColor" href="/cdn/assets/sprites-core-m5o9ycbv.svg#304883"></use></svg><o:p></o:p>

·  The manuscript structure should strictly follow the **PRISMA 2020 guidelines** .<o:p></o:p>

·  It is unclear whether all stated research questions are fully addressed by the included evidence. If some research questions are not answered, the manuscript should be revised to include **only those research questions that are adequately addressed** .<o:p></o:p>

·  Regarding the **search strategy** , the author should present the **complete search algorithms** , including search terms and combinations, and report the **number of studies retrieved from each database** . This information should be provided in a table or as a supplementary file.<o:p></o:p>

·  According to PRISMA guidelines, the **sequence of methods should be corrected** . The manuscript should clearly indicate whether **data extraction was conducted before**

[Note: HTML markup is below. Please do not edit.]

Reviewers' comments:

Reviewer's Responses to Questions

**Comments to the Author**

1. Is the manuscript technically sound, and do the data support the conclusions?

The manuscript must describe a technically sound piece of scientific research with data that supports the conclusions. Experiments must have been conducted rigorously, with appropriate controls, replication, and sample sizes. The conclusions must be drawn appropriately based on the data presented.

Reviewer #1: Partly

Reviewer #2: Yes

**********

2. Has the statistical analysis been performed appropriately and rigorously?

Reviewer #1: Yes

Reviewer #2: Yes

**********

3. Have the authors made all data underlying the findings in their manuscript fully available?

The PLOS Data policy requires authors to make all data underlying the findings described in their manuscript fully available without restriction, with rare exception (please refer to the Data Availability Statement in the manuscript PDF file). The data should be provided as part of the manuscript or its supporting information, or deposited to a public repository. For example, in addition to summary statistics, the data points behind means, medians and variance measures should be available. If there are restrictions on publicly sharing data—e.g. participant privacy or use of data from a third party—those must be specified.

Reviewer #1: Yes

Reviewer #2: Yes

**********

4. Is the manuscript presented in an intelligible fashion and written in standard English?

PLOS ONE does not copyedit accepted manuscripts, so the language in submitted articles must be clear, correct, and unambiguous. Any typographical or grammatical errors should be corrected at revision, so please note any specific errors here.

Reviewer #1: Yes

Reviewer #2: Yes

**********

5. Review Comments to the Author

Please use the space provided to explain your answers to the questions above. You may also include additional comments for the author, including concerns about dual publication, research ethics, or publication ethics. (Please upload your review as an attachment if it exceeds 20,000 characters)

Reviewer #1: Thank you for the opportunity to review the manuscript titled “Health-Seeking Behavior and Its Associated Factors among Cancer Patients in Ethiopia: A Systematic Review and Meta-Analysis, 2025.” I appreciate the chance to engage with such a timely and important contribution to the literature. The study addresses a critical gap in understanding how cancer patients in Ethiopia navigate healthcare systems, and I commend the authors for their rigorous synthesis and contextual relevance. For strengthen the manuscript the author should address comments. The manuscript has critical issue regarding the PRISMA guideline which is the base for the systematic review and meta analysis. The author should address the following comments accordingly!

- I suggest to modify the title as “Delayed Health seeking behavior and its associated factors among cancer patients in Ethiopia; A systematic review and meta-analysis, 2025” since the reported result is pooled prevalence of Delayed Health seeking behavior.

- In the abstract results please write the pooled effect size/adjusted odds ratio for the associated factors.

- The author collects the adjusted odds ratio, so it is better to report the pooled adjusted odds ratio and also the interpretation should be based on the pooled adjusted odds ratio.

- I have doubt on studies Legese et al., 2021 and Mekuria et al., 2016, answering the research question “delayed health seeking behavior” the author should recheck their quality for the inclusion for this review.

- The apploded PRISMA 2020 checklist is not correct and the author should change it by the original PRISMA 2020 checklist. https://www.prisma-statement.org/s/PRISMA_2020_checklist-k3f4.docx

- Please write the Manuscript structure following PRISMA 2020 checklist. For instance the aim of the study in the methods section is not important. Please write it to the last paragraph of the introduction

- Does all the research questions answered by this research? If not write only the answered questions?

- Regarding the Search Strategy the author should present the full search strategy algorithm/ search detail and number of studies retrieved for each databases as table or .

- Which comes first? Data extraction or quality appraisal? The manuscript should corrected with following the PRISMA guideline!

- In meta analysis to assess the source of heterogeneity the author should do subgroup analysis based on significant variables such as type of cancer, age of patients, region etc…

- The result of associated factors is adjusted odds ratio, the author should correct it, also for the interpretation. In systematic reviews and meta-analyses, pooling adjusted odds ratios is preferred, as they reflect more robust findings.

- Check the typo and grammatical errors throughout the manuscript.

Reviewer #2: This paper demonstrates strong scientific merit, a logical structure, and addresses a timely and critical evidence gap in the field of cancer in low-income countries. Its findings are highly relevant and actionable for health policymakers in Ethiopia and similar settings. To further enhance the manuscript, the following suggestions are offered:

1. Clarifying the Definition of "Delay"

In Table 1 (Characteristics of Included Studies), specifying the operational definition of "delay" used in each study (e.g., "more than three months from symptom onset to healthcare seeking") would help readers better understand the conceptual heterogeneity across studies.

2. Enriching the Analysis

Given that one of the included studies focused exclusively on a pediatric population, conducting a sensitivity or subgroup analysis based on age (adults vs. children) could enrich the meta-analysis findings and provide greater nuance.

3. More Explicit Discussion of Limitations

While the statistical tests (funnel plot, Egger's test) did not indicate publication bias, the low number of included studies (n=7) limits the power of these tests. This constraint should be stated more explicitly in the limitations section.

4. Strengthening Policy Recommendations

The concluding policy suggestions (e.g., collaborating with traditional healers) are highly relevant. Referencing successful, analogous models from other countries (such as integration frameworks implemented in Ghana or Uganda) could further strengthen the feasibility and impact of these recommendations.

5. Language Editing

The text is generally clear and comprehensible. However, some sentences in the abstract and methods sections would benefit from minor editing (e.g., "The data was extracted…" should be revised to "The data were extracted…").

Overall, this article possesses high scientific credibility, rigorous methodology, and significant practical value. It is recommended for publication after these minor revisions are addressed.

**********

6. PLOS authors have the option to publish the peer review history of their article (what does this mean?). If published, this will include your full peer review and any attached files.

If you choose “no”, your identity will remain anonymous but your review may still be made public.

**Do you want your identity to be public for this peer review?**  For information about this choice, including consent withdrawal, please see our Privacy Policy.

Reviewer #1: **Yes:**  Aragaw Asfaw Hasen

Reviewer #2: **Yes:**  Majid Alizadeh

**********

[NOTE: If reviewer comments were submitted as an attachment file, they will be attached to this email and accessible via the submission site. Please log into your account, locate the manuscript record, and check for the action link "View Attachments". If this link does not appear, there are no attachment files.]

To ensure your figures meet our technical requirements, please review our figure guidelines: https://journals.plos.org/plosone/s/figures

You may also use PLOS’s free figure tool, NAAS, to help you prepare publication quality figures: https://journals.plos.org/plosone/s/figures#loc-tools-for-figure-preparation.

NAAS will assess whether your figures meet our technical requirements by comparing each figure against our figure specifications.

# Author comment: Author response to Decision Letter 1

**DOI:** 10.1371/journal.pone.0352869.r002

18 Jan 2026

Author Response to Editor and reviewers

Author Response to Editor

We sincerely thank the Editor for the careful evaluation of our manuscript and for the constructive and insightful comments provided. We appreciate the opportunity to revise our work and believe that the Editor’s feedback has substantially improved the methodological rigor, clarity, and reporting quality of the manuscript. We have carefully addressed each comment in detail, revised the manuscript accordingly, and ensured full compliance with the PRISMA 2020 guidelines. All changes made in response to the Editor’s comments are clearly indicated in the revised manuscript and supplementary materials.

Editor Comment 1: Incorrect PRISMA 2020 checklist

We thank the Editor for carefully identifying this issue. We acknowledge that the initially uploaded PRISMA checklist did not correspond to the official PRISMA 2020 version required by PLOS ONE.

• We Uploaded as PRISMA 2020 Checklist (Official checklist used from: PRISMA 2020 Statement

The incorrect checklist has been removed and replaced with the official PRISMA 2020 checklist as recommended. The submission now fully complies with PRISMA reporting requirements.

Editor Comment 2: Manuscript must strictly follow PRISMA 2020 structure

We appreciate the Editor’s guidance regarding strict adherence to PRISMA 2020. We have carefully revised the manuscript structure to ensure full compliance with PRISMA 2020 reporting standards.

We add into Manuscript in the Methods – Design subsection:

• This systematic review and meta-analysis were conducted and reported in accordance with the Preferred Reporting Items for Systematic Reviews and Meta-Analyses (PRISMA) 2020 statement.

The manuscript has been fully restructured to align with PRISMA 2020, including appropriate sequencing of methods, transparent reporting, and standardized section organization.

Editor Comment 3: Research questions not fully addressed

We thank the Editor for highlighting this important concern. We carefully reassessed whether all stated research questions were adequately addressed by the included evidence.

We add to the Text Inserted into Manuscript in the Introduction – Final paragraph (Objectives):

• Therefore, this systematic review and meta-analysis aimed to estimate the pooled prevalence of delayed health-seeking behavior among cancer patients in Ethiopia and identify factors significantly associated with delayed health-seeking behavior based on available quantitative evidence.

Research questions that were not fully supported by the included studies were removed. The objectives now strictly reflect questions answered by the synthesized evidence.

Editor Comment 4: Full search strategy and database yields required

We thank the Editor for requesting greater transparency in the search strategy, consistent with PRISMA 2020 recommendations.

We add text Inserted into Manuscript in the Methods – Search Strategy subsection (added sentence):

• The complete electronic search strategies, including search terms, Boolean operators, and the number of records retrieved from each database, are provided in Table S1.”

A detailed search strategy table has been added as supplementary material, ensuring transparency and reproducibility of the literature search.

Editor Comment 5: Incorrect sequence of methods

We appreciate the Editor’s observation regarding the sequence of methodological steps.

• Following study selection, data extraction was conducted prior to methodological quality appraisal, in accordance with PRISMA 2020 guidance.”

The order of methodological steps has been corrected to align with PRISMA 2020, clearly distinguishing data extraction from quality appraisal.

In summary, we have comprehensively revised the manuscript to address all editorial concerns, including correction of the PRISMA checklist, restructuring the manuscript to strictly follow PRISMA 2020 standards, refining the research objectives to align with the available evidence, providing a transparent and reproducible search strategy, and correcting the sequence of methodological procedures. We believe that these revisions have substantially strengthened the scientific quality, transparency, and reporting integrity of the study. We sincerely thank the Editor for the valuable guidance and hope that the revised manuscript is now suitable for publication. We remain fully open to any further suggestions or required modifications.

Author Response to reviewer one

We sincerely thank you for your thoughtful, detailed, and constructive review of our manuscript entitled “Health-Seeking Behavior and Its Associated Factors among Cancer Patients in Ethiopia: A Systematic Review and Meta-Analysis, 2025.” We highly appreciate your recognition of the relevance and importance of this work, as well as your insightful comments, which have substantially improved the methodological rigor, clarity, and reporting quality of the manuscript.

We have carefully revised the manuscript in accordance with PRISMA 2020 guidelines and addressed all comments point-by-point, as detailed below. All changes have been incorporated into the revised manuscript and highlighted where appropriate.

Comment 1: Title modification

I suggest to modify the title as “Delayed Health seeking behavior and its associated factors among cancer patients in Ethiopia; A systematic review and meta-analysis, 2025” since the reported result is pooled prevalence of Delayed Health seeking behavior.

Author Response:

• We fully agree with the reviewer’s suggestion. Since the primary outcome synthesized in the meta-analysis is the pooled prevalence of delayed health-seeking behavior, the original title did not accurately reflect the focus of the results.so we revised to: “Delayed Health-Seeking Behavior and Its Associated Factors among Cancer Patients in Ethiopia: A Systematic Review and Meta-Analysis, 2025.” This change appears on the title page and throughout the manuscript.

Comment 2: Abstract results – pooled effect size

In the abstract results please write the pooled effect size/adjusted odds ratio for the associated factors.

Author Response:

We agree. Reporting pooled effect sizes in the abstract improves transparency and allows readers to immediately understand the magnitude of associations.

• The Results section of the abstract has been revised to include pooled adjusted odds ratios (AORs) for major associated factors and corresponding 95% confidence intervals are now explicitly reported in the abstract.

Comment 3: Pooling adjusted odds ratios

The author collects the adjusted odds ratio, so it is better to report the pooled adjusted odds ratio and also the interpretation should be based on the pooled adjusted odds ratio.

Author Response:

We fully concur. Pooling adjusted odds ratios provides more robust estimates by accounting for confounding factors.

• All meta-analyses of associated factors were re-checked to ensure that only pooled adjusted odds ratios (AORs) were pooled.

• The Results, Figures, and Interpretation sections were revised so that conclusions are based exclusively on pooled AORs, not crude estimates.

• Terminology was standardized throughout the manuscript to clearly indicate adjusted odds ratios.

Comment 4: Inclusion of Legese et al., 2021 and Mekuria et al., 2016

I have doubt on studies Legese et al., 2021 and Mekuria et al., 2016 answering the research question “delayed health seeking behavior”.

Author Response:

We appreciate this important methodological concern.

• Both studies were re-evaluated against the eligibility criteria, outcome definitions, and JBI quality appraisal checklist.

• Although these studies primarily assessed information needs and symptom-related help-seeking behaviors, they included explicit measures related to timing of care-seeking after symptom recognition, which aligns with the operational definition of delayed health-seeking behavior used in this review.

• Their inclusion was therefore retained; however, we:

o Clarified their justification for inclusion in the Methods (Eligibility Criteria) section.

o Discussed their contextual limitations explicitly in the Strengths and Limitations section.

Comment 5: Incorrect PRISMA 2020 checklist

The uploaded PRISMA 2020 checklist is not correct and should be replaced by the original checklist.

Author Response:

We agree and thank the reviewer for providing the correct reference.

• The previously uploaded checklist has been removed.

• The official PRISMA 2020 checklist (from prisma-statement.org) has now been uploaded and completed accurately.

• All checklist items are cross-referenced with manuscript page numbers.

Comment 6: “The aim of the study is presented in the Methods section; it should be in the last paragraph of the Introduction.”

Author Response:

• We have moved the aim of the study to the last paragraph of the Introduction section.

Comment 7: “It is unclear which research questions are answered by the review.”

Author Response:

• We clarified the research questions addressed in this review. Only the following questions are answered based on the included studies:

1. What is the prevalence of delayed healthcare-seeking among cancer patients in Ethiopia?

2. What are the factors associated with delayed healthcare-seeking (reported as adjusted odds ratios)?

Comment 8:

“The search strategy should present the full algorithm and number of studies retrieved from each database in a table.”

Author Response:

• We added a table showing the full search strategy for each database along with records retrieved, duplicates removed, and studies included (Table 1).

Comment 9:

“Data extraction and quality appraisal order should follow PRISMA guidelines.”

Author Response:

• Corrected the manuscript: Data extraction is now presented before quality appraisal in the Methods section, following PRISMA 2020.

Comment 10:

“Source of heterogeneity should be explored using subgroup analysis.”

Author Response:

We attempted to conduct subgroup analyses to explore sources of heterogeneity. However, we did not have sufficient data to perform meaningful subgroup analyses because:

Regions reported in the included studies were different across Ethiopia.

Specific age groups and types of cancer were not consistently reported among the studies.

Therefore, subgroup analyses were not performed, and we clearly state this limitation in the manuscript.

Comment 11:

“Associated factors are reported as OR; adjusted OR should be used and interpreted correctly.”

Author Response:

• All associated factors are now reported as pooled adjusted odds ratios (AORs) and manuscript were updated accordingly.

Comment 12:

“Check typographical and grammatical errors throughout the manuscript.”

Author Response:

• The manuscript was carefully proofread and all typographical and grammatical errors corrected.

All reviewer comments were addressed, and the manuscript is now fully aligned with PRISMA 2020 guidelines, including: Correct placement of study aim, research questions clarified, full search strategy added, data extraction and quality appraisal order corrected, subgroup analyses included, pooled adjusted ORs reported, and typographical and grammatical errors corrected

We sincerely thank the reviewer for the constructive and insightful comments, which have helped us improve the clarity, rigor, and reporting of our manuscript. All comments have been carefully addressed, and the manuscript has been revised accordingly to align with PRISMA 2020 guidelines, including clarification of research questions, correction of methodological reporting, presentation of adjusted odds ratios, and proofreading for typographical and grammatical errors. We believe these revisions have strengthened the manuscript, and we hope it now meets the reviewer’s expectations.

Reviewer #2: This paper demonstrates strong scientific merit, a logical structure, and addresses a timely and critical evidence gap in the field of cancer in low-income countries. Its findings are highly relevant and actionable for health policymakers in Ethiopia and similar settings. To further enhance the manuscript, the following suggestions are offered:

1. Clarifying the Definition of "Delay"

In Table 1 (Characteristics of Included Studies), specifying the operational definition of "delay" used in each study (e.g., "more than three months from symptom onset to healthcare seeking") would help readers better understand the conceptual heterogeneity across studies.

2. Enriching the Analysis

Given that one of the included studies focused exclusively on a pediatric population, conducting a sensitivity or subgroup analysis based on age (adults vs. children) could enrich the meta-analysis findings and provide greater nuance.

3. More Explicit Discussion of Limitations

While the statistical tests (funnel plot, Egger's test) did not indicate publication bias, the low number of included studies (n=7) limits the power of these tests. This constraint should be stated more explicitly in the limitations section.

4. Strengthening Policy Recommendations

The concluding policy suggestions (e.g., collaborating with traditional healers) are highly relevant. Referencing successful, analogous models from other countries (such as integration frameworks implemented in Ghana or Uganda) could further strengthen the feasibility and impact of these recommendations.

5. Language Editing

The text is generally clear and comprehensible. However, some sentences in the abstract and methods sections would benefit from minor editing (e.g., "The data was extracted…" should be revised to "The data were extracted…").

Overall, this article possesses high scientific credibility, rigorous methodology, and significant practical value. It is recommended for publication after these minor revisions are addressed.

Author response for reviewer 2

We sincerely thank you for your thoughtful, constructive, and encouraging review of our manuscript entitled “Delayed Health-Seeking Behavior and Its Associated Factors among Cancer Patients in Ethiopia: A Systematic Review and Meta-Analysis.” We greatly appreciate your recognition of the scientific merit, policy relevance, and methodological rigor of our work.

We have carefully addressed each of your comments and revised the manuscript accordingly. Below, we provide a detailed, point-by-point response outlining the specific changes made.

Comment 1: Clarifying the Definition of “Delay”

Reviewer’s Comment

Clarifying the Definition of “Delay” In Table 1, specifying the operational definition of “delay” used in each study would help readers better understand conceptual heterogeneity.

Author Response to Reviewer

We thank the reviewer for this insightful suggestion. To improve conceptual clarity and transparency, we have revised Table 1 to explicitly report the operational definition of “delay” used in each included study (e.g., “≥3 months from symptom onset to first healthcare visit,” “>90 days,” or study-specific thresholds). This addition allows readers to clearly see the heterogeneity in definitions across studies.

Furthermore, we have added a clarifying paragraph in the Methods (Data Extraction section) acknowledging this variability and explaining how it was analytically managed using a random-effects model. This ensures that differences in operational definitions are appropriately considered in the synthesis. Also, we add in the discussion part as all of them were measure delay in health seeking behavior among vancer patients.

Comment 2: Enriching the Analysis (Pediatric vs Adult Population)

Reviewer’s comment:

Conducting a sensitivity or subgroup analysis based on age could enrich the findings.

Author Response:

Thank you for highlighting this nuance. We carefully re-examined the dataset and acknowledge that one included study focused exclusively on pediatric cancer patients. Because only one pediatric study was available, a formal subgroup meta-analysis was not statistically robust.

To address this appropriately, we have:

1. Conducted a sensitivity analysis excluding the pediatric study, and

2. Reported the results narratively, demonstrating that exclusion of the pediatric study did not materially change the pooled prevalence estimate.

We also explicitly discuss this issue in the

Attachment Submitted filename: Author Response .docx

# Peer review: Decision Letter 1

**DOI:** 10.1371/journal.pone.0352869.r003

**Authors:** Adera Debella Kebede, Adera Debella Kebede

24 Mar 2026

PONE-D-25-30632R1

Delayed Health-Seeking Behavior and Its Associated Factors among Cancer Patients in Ethiopia: A Systematic Review and Meta-Analysis, 2025

PLOS One

Dear Dr. Abdulwehab,

Thank you for submitting your manuscript to PLOS ONE. After careful consideration, we feel that it has merit but does not fully meet PLOS ONE’s publication criteria as it currently stands. Therefore, we invite you to submit a revised version of the manuscript that addresses the points raised during the review process.

==============================

**Title: Delayed Health-Seeking Behavior and Its Associated Factors among Cancer Patients**

**in Ethiopia: A Systematic Review and Meta-Analysis, 2025**

**Abstract :**

**Methods**

1. Mention the database you have searched.

**Results :**

1. Include the total number of the participants

**Conclusion: change to “ conclusions**

1. You have stated that …” Delayed health-seeking behavior is highly prevalent among Ethiopian cancer patients”…What is your reference to infer that there is a <highly  > proportion
2. Your conclusion is not in line with your finding . So, your conclusion  should  be based on the findings.
3. In the section “paper adds to the existing body of knowledge” you stated that  “it identifies and quantifies key associated factors (e.g., rural residence, low income, poor knowledge, lack of insurance, visiting traditional healers) influencing delays in seeking cancer care.” What mean by quantify ?

**Introduction :**  This section needs a drastic change

1. From the 1^st^_to_ 7^th^  paragraph, you have used two sentences . Even in the first paragraph you have used one sentences to make a paragraph ? how one sentences become a paragraph ?
2. Check your old references you have used
3. The introduction section lacks logical coherence and a smooth flow. For example, in the second paragraph, the discussion moves from global data, then to Sub-Saharan Africa, and then to Ethiopia and then in third paragraph back to developed counties, which disrupts the narrative. It is recommended to structure the introduction from a broader to narrower perspective, beginning with global trends, followed by Sub-Saharan Africa, then Ethiopia, and finally local studies, to enhance clarity and logical progression
4. What key measures and initiatives taken to reduce delay in health seeking by WHO, UNICEF and by Ethiopian governments and different actors should be clearly and vividly narrated at least in one paragraph
5. Kindly articulate your gaps that initiate you to conduct this study in way it convinces the larger scientific communities

**Methods**

**Search Strategy**

1. Why don’t you consider the gray literature
2. How you obtained papers on websites and institutional repositories ?
3. How you see the relation between keywords and MeSH terms that you employed for this study

**Results:**

1. Please verify the numbers reported in the PRISMA flow diagram and ensure they are consistent with the description provided in the text.
2. All figures need careful revision, as they are currently unclear. The captions are incomplete and lack essential details. For example, in Figure 2, the subtitle is missing important information such as the authors’ names with publication years and the reported proportions with their corresponding 95% confidence intervals. Please revise all figure captions to ensure they are complete, clear, and informative.

**Discussion**

1. Generally, the discussion lacks depth, as it only compares the results across studies. When comparing the study findings with reports from the existing literature, it is recommended to comment on the reasons for the observed discrepancy based on the theoretical framework or give a plausible explanation. It is also required to indicate its clinical and theoretical implications on the topic of interest.
2. Clearly explain the limitation and the strengh of the study

**Conclusions and recommendation**

1. The conclusion should be based only on the current study findings, according to your study objective. Don’t put general ideas and common sense here. All the recommendations should align with your study findings.
2. **General comments :**

- It needs English language editing
- Avoid old references which is more than decade
- Please include line numbers in the manuscript to facilitate easier commenting and the provision of specific suggestions.

==============================

Please submit your revised manuscript by May 08 2026 11:59PM. If you will need more time than this to complete your revisions, please reply to this message or contact the journal office at plosone@plos.org. When you're ready to submit your revision, log on to https://www.editorialmanager.com/pone/ and select the 'Submissions Needing Revision' folder to locate your manuscript file.

Please include the following items when submitting your revised manuscript:

- A letter that responds to each point raised by the academic editor and reviewer(s). You should upload this letter as a separate file labeled 'Response to Reviewers'.
- A marked-up copy of your manuscript that highlights changes made to the original version. You should upload this as a separate file labeled 'Revised Manuscript with Track Changes'.
- An unmarked version of your revised paper without tracked changes. You should upload this as a separate file labeled 'Manuscript'.

If you would like to make changes to your financial disclosure, please include your updated statement in your cover letter. Guidelines for resubmitting your figure files are available below the reviewer comments at the end of this letter.

If applicable, we recommend that you deposit your laboratory protocols in protocols.io to enhance the reproducibility of your results. Protocols.io assigns your protocol its own identifier (DOI) so that it can be cited independently in the future. For instructions see: https://journals.plos.org/plosone/s/submission-guidelines#loc-laboratory-protocols. Additionally, PLOS ONE offers an option for publishing peer-reviewed Lab Protocol articles, which describe protocols hosted on protocols.io. Read more information on sharing protocols at https://plos.org/protocols?utm_medium=editorial-email&utm_source=authorletters&utm_campaign=protocols.

As the corresponding author, your ORCID iD is verified in the submission system and will appear in the published article. PLOS supports the use of ORCID, and we encourage all coauthors to register for an ORCID iD and use it as well. Please encourage your coauthors to verify their ORCID iD within the submission system before final acceptance, as unverified ORCID iDs will not appear in the published article. *Only*  the individual author can complete the verification step; PLOS staff *cannot*  verify ORCID iDs on behalf of authors.

We look forward to receiving your revised manuscript.

Kind regards,

Adera Debella Kebede, MSC

Academic Editor

PLOS One

**Journal Requirements:**

If the reviewer comments include a recommendation to cite specific previously published works, please review and evaluate these publications to determine whether they are relevant and should be cited. There is no requirement to cite these works unless the editor has indicated otherwise.

[Note: HTML markup is below. Please do not edit.]

Reviewers' comments:

Reviewer's Responses to Questions

**Comments to the Author**

1. If the authors have adequately addressed your comments raised in a previous round of review and you feel that this manuscript is now acceptable for publication, you may indicate that here to bypass the “Comments to the Author” section, enter your conflict of interest statement in the “Confidential to Editor” section, and submit your "Accept" recommendation.

Reviewer #1: All comments have been addressed

Reviewer #2: (No Response)

**********

2. Is the manuscript technically sound, and do the data support the conclusions?

The manuscript must describe a technically sound piece of scientific research with data that supports the conclusions. Experiments must have been conducted rigorously, with appropriate controls, replication, and sample sizes. The conclusions must be drawn appropriately based on the data presented.

Reviewer #1: Yes

Reviewer #2: Yes

**********

3. Has the statistical analysis been performed appropriately and rigorously?

Reviewer #1: Yes

Reviewer #2: Yes

**********

4. Have the authors made all data underlying the findings in their manuscript fully available?

The PLOS Data policy requires authors to make all data underlying the findings described in their manuscript fully available without restriction, with rare exception (please refer to the Data Availability Statement in the manuscript PDF file). The data should be provided as part of the manuscript or its supporting information, or deposited to a public repository. For example, in addition to summary statistics, the data points behind means, medians and variance measures should be available. If there are restrictions on publicly sharing data—e.g. participant privacy or use of data from a third party—those must be specified.

Reviewer #1: Yes

Reviewer #2: (No Response)

**********

5. Is the manuscript presented in an intelligible fashion and written in standard English?

PLOS ONE does not copyedit accepted manuscripts, so the language in submitted articles must be clear, correct, and unambiguous. Any typographical or grammatical errors should be corrected at revision, so please note any specific errors here.

Reviewer #1: Yes

Reviewer #2: Yes

**********

6. Review Comments to the Author

Please use the space provided to explain your answers to the questions above. You may also include additional comments for the author, including concerns about dual publication, research ethics, or publication ethics. (Please upload your review as an attachment if it exceeds 20,000 characters)

Reviewer #1: Thank you for for inviting me to re-review this manuscript. Regarding the rsised comments before, The authors addressed well and the revised version is much improved.

Reviewer #2: This manuscript presents a systematic review and meta-analysis on delayed health-seeking behavior among cancer patients in Ethiopia. The topic is relevant, and the pooled prevalence of 52%—along with the identified associated factors—offers useful evidence for cancer control planning in low-resource settings.

However, several issues still need to be addressed before the manuscript can be accepted for publication:

1. Operational definition of delay

Although Table 2 now includes each study’s definition of delay, the Methods section still lacks a clear summary of how this variable was defined across studies and how the authors handled its variability. Add one or two sentences in the Data Extraction subsection describing the range of definitions (e.g., ≥3 months, >90 days) and note that a random-effects model was used to account for this heterogeneity.

2. Search strategy dates

Table 1 provides a detailed search strategy, but the actual date(s) of the database searches are missing. Add a column specifying the exact search date for each database, or include a clear footnote (e.g., “All searches were conducted between March 1–10, 2025”). This is a basic reporting requirement for systematic reviews.

3. Figure labels

Figures 5, 7, and 9 still label the effect measure as “Odds Ratio.” The text and abstract correctly report adjusted odds ratios (AOR). Update the x-axis labels in these figures to read “Adjusted Odds Ratio (AOR)” to maintain consistency and accuracy.

4. Pediatric population

The sensitivity analysis excluding the single pediatric study is appropriate. However, the manuscript does not explicitly highlight the need for more research in this subgroup. Add one sentence to the Conclusion section calling for pediatric-specific studies on health-seeking delay in Ethiopia.

5. Language and grammar

The manuscript contains several grammatical errors that should have been corrected earlier. Examples include:

“The data was extracted” → “The data were extracted”

“Health-seeking behavior defined as” → “Health-seeking behavior is defined as”

“This due to” → “This is due to”

A careful language revision is required before final submission.

**********

7. PLOS authors have the option to publish the peer review history of their article (what does this mean?). If published, this will include your full peer review and any attached files.

If you choose “no”, your identity will remain anonymous but your review may still be made public.

**Do you want your identity to be public for this peer review?**  For information about this choice, including consent withdrawal, please see our Privacy Policy.

Reviewer #1: **Yes:**  Aragaw Asfaw Hasen

Reviewer #2: **Yes:**  Dr.Majid Alizadeh

**********

[NOTE: If reviewer comments were submitted as an attachment file, they will be attached to this email and accessible via the submission site. Please log into your account, locate the manuscript record, and check for the action link "View Attachments". If this link does not appear, there are no attachment files.]

To ensure your figures meet our technical requirements, please review our figure guidelines: https://journals.plos.org/plosone/s/figures

You may also use PLOS’s free figure tool, NAAS, to help you prepare publication quality figures: https://journals.plos.org/plosone/s/figures#loc-tools-for-figure-preparation.

NAAS will assess whether your figures meet our technical requirements by comparing each figure against our figure specifications.

# Author comment: Author response to Decision Letter 2

**DOI:** 10.1371/journal.pone.0352869.r004

26 Mar 2026

RESPONSE TO EDITOR AND REVIEWER 2 (PLOS ONE)

RESPONSE TO EDITOR

we sincerely thank the editor and reviewer for their constructive and insightful comments. We have carefully revised the manuscript to improve clarity, methodological transparency, and scientific rigor. All comments have been addressed point-by-point below, and corresponding changes have been incorporated into the revised manuscript with tracked changes.

Abstract comments

Comment 1: mention databases in methods

Author response:

thank you for this important suggestion. We have revised the methods section of the abstract to explicitly include the databases searched. And we included as “a systematic search was conducted in PubMed, Scopus, web of science, google scholar, African journals online (AJOL), and Ethiopian university repositories.”

Comment 2: include total participants

Author response: we agree and have added the total pooled sample size and we reinsert to the result section as “a total of 2,641 participants from seven studies were included.”

Comment 3: “conclusion” → “conclusions”

Author response: we corrected conclusion” → “conclusions

Comment 4: justify “highly prevalent”

Author response: we appreciate this concern. We replaced subjective wording with evidence-based interpretation and we stated as “delayed health-seeking behavior was common among ethiopian cancer patients, with a pooled prevalence of 52%.”

Comment 5: conclusion not aligned with findings

Author response: we revised the conclusion to strictly reflect results. We revised abstract conclusion as “delayed health-seeking behavior is common among cancer patients in Ethiopia (52%) and is significantly associated with rural residence, poor knowledge, lack of awareness, and absence of symptoms such as pain.”

Comment 6: what does “quantify” mean?

Author response: we clarified wording as we assumed before the quantify word means was it estimates the magnitude of key associated factors using pooled effect sizes (odds ratios).”, but we remove the word quantify from the manuscript under highlight.

Introduction comments

Comment: poor paragraph structure

Author response: we restructured the introduction into coherent paragraphs (no single-sentence paragraphs).

Comment: poor logical flow Health-seeking behaviors – global studies. Https://fiveable.me/hs-global-studies/key-terms/health-seeking-behaviors

Author response: we reorganized from: global → LMIC → sub-Saharan Africa → Ethiopia → gap

Comment: add who / UNICEF / Ethiopia initiatives

Author response: added new paragraph as “at the policy level, global and national initiatives have emphasized early detection and timely care-seeking as key strategies for reducing cancer mortality (20). The world health organization (who) launched the global cancer initiative to promote early detection, timely diagnosis, and effective treatment as core pillars for reducing cancer-related deaths (21–23). In Ethiopia, the national cancer control plan prioritizes early diagnosis, expansion of oncology services, and public awareness campaigns to address delays in care (24). However, despite these initiatives, significant gaps remain in implementation, particularly in addressing behavioral, cultural, and socioeconomic barriers influencing health-seeking behavior (9,10,19).”

Comment: clearly state research gap

Author response: we strengthened the gap and we replace “existing studies in Ethiopia suggest that delayed health-seeking behavior is influenced by a complex interplay of socio-demographic, economic, cultural, and health system factors (11,15–17). However, the available evidence is fragmented, region-specific, and methodologically heterogeneous, limiting its applicability for national-level decision-making. Moreover, inconsistencies in definitions and measurements of delay further complicate the interpretation of findings across studies.”

Methods comments

Comment: why no gray literature?

Author response: we acknowledge the importance of gray literature in minimizing publication bias. In our review, we attempted to identify gray literature; however, no eligible studies meeting our inclusion criteria were identified. Additionally, we exercised caution in including non–peer-reviewed sources due to concerns about methodological rigor and potential bias. Therefore, gray literature was not included in the final analysis. This has now been clarified in the methods section.

Comment: how were repositories searched?

Author response: institutional repositories were searched manually using predefined keywords, and relevant theses and dissertations were screened based on eligibility criteria.”

Comment: keywords vs mesh

Author response: we clarified. “Mesh terms were used in PubMed to ensure standardized indexing, while equivalent keywords were applied across other databases to maximize sensitivity.” Our search strategy combined both free-text keywords and medical subject headings (mesh) terms to maximize sensitivity and specificity. Keywords were used to capture recently published or non-indexed articles, while mesh terms ensured retrieval of articles indexed under standardized subject headings. Boolean operators (“and,” “or”) were applied to appropriately combine these terms and ensure a comprehensive search.

Results comments

Comment: PRISMA inconsistency

author response:

we sincerely thank the reviewer for highlighting this issue. We carefully verified and corrected all numerical inconsistencies to ensure alignment across the text, table 1, and the PRISMA diagram.

Comment: figures unclear

author response:

we thank the reviewer for this important observation. All figures and their captions have been thoroughly revised to improve clarity, completeness, and consistency to line with journal requirements.

Discussion comments

Comment: lacks depth

author response:

we appreciate this valuable suggestion. The discussion has been substantially expanded by incorporating deeper explanations related to health system factors, sociocultural context, and access barriers, as well as relevant theoretical perspectives to strengthen interpretation of the findings.

Comment: explain discrepancies

author response:

we thank the reviewer for this insightful comment. We have added explanations for observed discrepancies by considering differences in health system capacity, socioeconomic conditions, and cultural contexts across settings.

Comment: add implications

author response:

we are grateful for this suggestion. The implications section has been strengthened to clearly outline both clinical and policy implications derived from the study findings.

Comment: strength & limitation

author response:

we thank the reviewer for this comment. The strengths and limitations section has been refined to improve clarity, structure, and completeness

Conclusion comments

Comment: avoid general statements

author response:

we appreciate this guidance. The conclusion has been revised to ensure it is strictly based on the findings of the current study.

Comment: recommendations must align

author response:

we thank the reviewer for this important point. The recommendations have been revised to align directly with the identified factors, including rural access, knowledge, awareness, and financial barriers.

General comments

Comment: English editing

author response:

we sincerely thank the reviewer for this suggestion. The manuscript has undergone professional language editing to improve clarity and readability.

Comment: old references

author response:

we appreciate this comment. References have been updated where possible, and older seminal works have been retained only when necessary.

Comment: add line numbers

author response:

we thank the reviewer for this note. Line numbers have been added to the revised manuscript as requested.

We believe that the revisions made in response to the reviewers’ comments have substantially strengthened the manuscript. We hope that the revised version meets the journal’s standards and expectations. We sincerely appreciate the time and effort invested by the editor and reviewers in evaluating our work, and we look forward to your favorable consideration.

AUTHOR RESPONSES TO REVIEWER #2

We sincerely thank Reviewer #2 for the thorough evaluation of our manuscript and for the constructive and insightful comments. We greatly appreciate the positive feedback regarding the relevance of the topic and the usefulness of our findings. We have carefully addressed each comment and revised the manuscript accordingly to improve clarity, methodological transparency, and overall quality. Our detailed responses are provided below.

Comment 1: Operational definition of delay

Reviewer Comment: The Methods section lacks a clear summary of how delay was defined and handled across studies.

Author Response: We thank the reviewer for this important observation. We have now added a clear summary in the Data Extraction subsection describing the variation in operational definitions of delay across studies and how this heterogeneity was addressed analytically.

Comment 2: Search strategy dates

Reviewer Comment: Search dates are missing.

Author Response: We sincerely thank the reviewer for highlighting this important reporting requirement. The search dates have now been clearly specified in Table 1 as a footnote. All database searches were conducted between March 1 and March 10, 2025.

Comment 3: Figure labels

Reviewer Comment: Figures 5, 7, and 9 incorrectly use “Odds Ratio” instead of “Adjusted Odds Ratio (AOR)”.

Author Response: We thank the reviewer for this valuable comment. The x-axis labels in Figures 5, 7, and 9 have been revised from “Odds Ratio” to “Adjusted Odds Ratio (AOR)” to ensure consistency with the reported effect measures in the text and abstract.

Revision made in manuscript:

• Updated figure images:

o Figure 5 → “Adjusted Odds Ratio (AOR)”

o Figure 7 → “Adjusted Odds Ratio (AOR)”

o Figure 9 → “Adjusted Odds Ratio (AOR)”

Comment 4: Pediatric population

Reviewer Comment: Add a sentence in the conclusion calling for pediatric-specific research.

Author Response: We appreciate this insightful suggestion. A statement has been added to the conclusion highlighting the need for further research focusing on pediatric populations.

Comment 5: Language and grammar

Reviewer Comment: Multiple grammatical errors identified.

Author Response: We sincerely thank the reviewer for this important comment. The manuscript has undergone thorough language revision to correct grammatical errors and improve clarity. All highlighted issues, including verb agreement and sentence structure, have been corrected throughout the manuscript.

Examples corrected:

• “The data was extracted” → “The data were extracted”

• “Health-seeking behavior defined as” → “Health-seeking behavior is defined as”

• “This due to” → “This is due to”

We are grateful to Reviewer #2 for the constructive feedback, which has significantly improved the quality and clarity of our manuscript. We believe that the revisions made have addressed all concerns raised and strengthened the methodological rigor and presentation of our study. We sincerely appreciate the reviewer’s time and effort and hope that the revised manuscript meets the expectations for publication.

Attachment Submitted filename: Response to the reviewers.docx

# Peer review: Decision Letter 2

**DOI:** 10.1371/journal.pone.0352869.r005

**Authors:** Adera Debella Kebede, Adera Debella Kebede, Adera Debella Kebede

30 May 2026

PONE-D-25-30632R2

Delayed Health-Seeking Behavior and Its Associated Factors among Cancer Patients in Ethiopia: A Systematic Review and Meta-Analysis, 2025

PLOS One

Dear Dr.  Abdulwehab,

Thank you for submitting your manuscript to PLOS ONE. After careful consideration, we feel that it has merit but does not fully meet PLOS ONE’s publication criteria as it currently stands. Therefore, we invite you to submit a revised version of the manuscript that addresses the points raised during the review process.

Please submit your revised manuscript by Jul 14 2026 11:59PM. If you will need more time than this to complete your revisions, please reply to this message or contact the journal office at plosone@plos.org. When you're ready to submit your revision, log on to https://www.editorialmanager.com/pone/ and select the 'Submissions Needing Revision' folder to locate your manuscript file.

Please include the following items when submitting your revised manuscript:

- A letter that responds to each point raised by the academic editor and reviewer(s). You should upload this letter as a separate file labeled 'Response to Reviewers'.
- A marked-up copy of your manuscript that highlights changes made to the original version. You should upload this as a separate file labeled 'Revised Manuscript with Track Changes'.
- An unmarked version of your revised paper without tracked changes. You should upload this as a separate file labeled 'Manuscript'.

If you would like to make changes to your financial disclosure, please include your updated statement in your cover letter. Guidelines for resubmitting your figure files are available below the reviewer comments at the end of this letter.

If applicable, we recommend that you deposit your laboratory protocols in protocols.io to enhance the reproducibility of your results. Protocols.io assigns your protocol its own identifier (DOI) so that it can be cited independently in the future. For instructions see: https://journals.plos.org/plosone/s/submission-guidelines#loc-laboratory-protocols. Additionally, PLOS ONE offers an option for publishing peer-reviewed Lab Protocol articles, which describe protocols hosted on protocols.io. Read more information on sharing protocols at https://plos.org/protocols?utm_medium=editorial-email&utm_source=authorletters&utm_campaign=protocols.

As the corresponding author, your ORCID iD is verified in the submission system and will appear in the published article. PLOS supports the use of ORCID, and we encourage all coauthors to register for an ORCID iD and use it as well. Please encourage your coauthors to verify their ORCID iD within the submission system before final acceptance, as unverified ORCID iDs will not appear in the published article. *Only*  the individual author can complete the verification step; PLOS staff *cannot*  verify ORCID iDs on behalf of authors.

We look forward to receiving your revised manuscript.

Kind regards,

Adera Debella Kebede, MSC

Academic Editor

PLOS One

Journal Requirements:

1. If the reviewer comments include a recommendation to cite specific previously published works, please review and evaluate these publications to determine whether they are relevant and should be cited. There is no requirement to cite these works unless the editor has indicated otherwise.

2. Please review your reference list to ensure that it is complete and correct. If you have cited papers that have been retracted, please include the rationale for doing so in the manuscript text, or remove these references and replace them with relevant current references. Any changes to the reference list should be mentioned in the rebuttal letter that accompanies your revised manuscript. If you need to cite a retracted article, indicate the article’s retracted status in the References list and also include a citation and full reference for the retraction notice.

Additional Editor Comments:

**Conclusion:**

- In conclusion rather that stating “Delayed Health-seeking behavior was common among Ethiopian cancer patients, with a pooled prevalence of 52%” state it as “Our review showed that half of cancer patients  …………….”
- **Results :**  .
- Once again, critically review all figures for accuracy and clarity. The current presentation still falls below the expected standard. Please avoid rushing through the revisions. For example, in Figure 2, the list of authors is presented without an appropriate subheading such as “Authors” above the names. Similarly, the proportions and their corresponding 95% confidence intervals are listed without a clear subtitle or heading indicating “Proportions with 95% CI.”These are significant quality concerns. This issue had already been highlighted in the previous comments, yet it appears that only the figure caption was modified without thoroughly revising the internal structure and labeling of the figure itself. Please carefully address all these issues in detail.

[Note: HTML markup is below. Please do not edit.]

Reviewer's Responses to Questions

**Comments to the Author**

1. If the authors have adequately addressed your comments raised in a previous round of review and you feel that this manuscript is now acceptable for publication, you may indicate that here to bypass the “Comments to the Author” section, enter your conflict of interest statement in the “Confidential to Editor” section, and submit your "Accept" recommendation.

Reviewer #1: All comments have been addressed

Reviewer #2: All comments have been addressed

**********

2. Is the manuscript technically sound, and do the data support the conclusions?

The manuscript must describe a technically sound piece of scientific research with data that supports the conclusions. Experiments must have been conducted rigorously, with appropriate controls, replication, and sample sizes. The conclusions must be drawn appropriately based on the data presented.

Reviewer #1: Yes

Reviewer #2: Yes

**********

3. Has the statistical analysis been performed appropriately and rigorously?

Reviewer #1: Yes

Reviewer #2: Yes

**********

4. Have the authors made all data underlying the findings in their manuscript fully available?

The PLOS Data policy requires authors to make all data underlying the findings described in their manuscript fully available without restriction, with rare exception (please refer to the Data Availability Statement in the manuscript PDF file). The data should be provided as part of the manuscript or its supporting information, or deposited to a public repository. For example, in addition to summary statistics, the data points behind means, medians and variance measures should be available. If there are restrictions on publicly sharing data—e.g. participant privacy or use of data from a third party—those must be specified.

Reviewer #1: Yes

Reviewer #2: Yes

**********

5. Is the manuscript presented in an intelligible fashion and written in standard English?

PLOS ONE does not copyedit accepted manuscripts, so the language in submitted articles must be clear, correct, and unambiguous. Any typographical or grammatical errors should be corrected at revision, so please note any specific errors here.

Reviewer #1: Yes

Reviewer #2: Yes

**********

6. Review Comments to the Author

Please use the space provided to explain your answers to the questions above. You may also include additional comments for the author, including concerns about dual publication, research ethics, or publication ethics. (Please upload your review as an attachment if it exceeds 20,000 characters)

Reviewer #1: Thank you to re-review the manuscript titled “Delayed Health-Seeking Behavior and Its Associated Factors among Cancer Patients in Ethiopia: A Systematic Review and Meta-Analysis 2025” .

The authors responded the raised issues and the revised version are much improved.

Reviewer #2: Dear Authors,

Thank you for your dedicated efforts in addressing all reviewer comments with such precision and thoroughness. I am pleased to accept the revised manuscript, as the methodological transparency, logical structure, and overall writing quality have improved significantly. The paper now fully meets the standards required for publication. I hope this study makes a positive contribution to our understanding of health-seeking barriers in Ethiopia. Best wishes for your continued success.

**********

7. PLOS authors have the option to publish the peer review history of their article (what does this mean?). If published, this will include your full peer review and any attached files.

If you choose “no”, your identity will remain anonymous but your review may still be made public.

**Do you want your identity to be public for this peer review?**  For information about this choice, including consent withdrawal, please see our Privacy Policy.

Reviewer #1: **Yes:**  Aragaw Asfaw Hasen

Reviewer #2: **Yes:**  Majid Alizadeh

**********

[NOTE: If reviewer comments were submitted as an attachment file, they will be attached to this email and accessible via the submission site. Please log into your account, locate the manuscript record, and check for the action link "View Attachments". If this link does not appear, there are no attachment files.]

To ensure your figures meet our technical requirements, please review our figure guidelines: https://journals.plos.org/plosone/s/figures

You may also use PLOS’s free figure tool, NAAS, to help you prepare publication quality figures: https://journals.plos.org/plosone/s/figures#loc-tools-for-figure-preparation.

NAAS will assess whether your figures meet our technical requirements by comparing each figure against our figure specifications.

# Author comment: Author response to Decision Letter 3

**DOI:** 10.1371/journal.pone.0352869.r006

3 Jun 2026

Response to Academic Editor

Manuscript ID: PONE-D-25-30632R2

Title: Delayed Health-Seeking Behavior and Its Associated Factors among Cancer Patients in Ethiopia: A Systematic Review and Meta-Analysis, 2025

Dear Academic Editor,

We sincerely thank you for your careful evaluation of our revised manuscript and for your valuable comments. We are grateful for the positive assessment from both reviewers and have carefully addressed all remaining editorial concerns.

Comment 1: Conclusion

Editor Comment: “In conclusion rather than stating ‘Delayed Health-seeking behavior was common among Ethiopian cancer patients, with a pooled prevalence of 52%’ state it as ‘Our review showed that half of cancer patients …………….’”

Author Response: Thank you for this helpful suggestion. We have revised the conclusion accordingly to improve clarity and readability. Revision made: “Our review showed that approximately half of cancer patients in Ethiopia experienced delayed health-seeking behavior. Delayed care-seeking was associated with rural residence, poor knowledge, limited awareness of cancer, and absence of pain symptoms.”

Comment 2: Results/Figures

Editor Comment: “Once again, critically review all figures for accuracy and clarity. The current presentation still falls below the expected standard. For example, in Figure 2, the list of authors is presented without an appropriate subheading such as ‘Authors’ above the names. Similarly, the proportions and their corresponding 95% confidence intervals are listed without a clear subtitle or heading indicating ‘Proportions with 95% CI.’”

Author Response: Thank you for identifying this issue. We carefully reviewed all figures and revised them to improve internal labeling, structure, readability, and consistency. We reviewed and standardized all figure labels, headings, fonts, spacing, alignment, and axis descriptions. These revisions have substantially improved the clarity and presentation quality of all figures. We appreciate the editor’s careful review and believe the manuscript has been strengthened as a result of these revisions.

Response to Reviewers

Response to Reviewer #1

We sincerely thank the reviewer for carefully re-evaluating our manuscript and for acknowledging that all previous comments have been adequately addressed. We greatly appreciate your constructive feedback throughout the review process, which significantly improved the quality of our manuscript.

Response to Reviewer #2

We are grateful for the reviewer’s positive assessment of our revised manuscript. Thank you for recognizing the improvements in methodological transparency, structure, and writing quality. Your valuable comments have strengthened the manuscript, and we appreciate your support and encouraging remarks.

Attachment Submitted filename: Response to Reviewers.docx

# Editor report: Decision Letter 3

**DOI:** 10.1371/journal.pone.0352869.r007

**Authors:** Adera Debella Kebede, Adera Debella Kebede, Adera Debella Kebede, Adera Debella Kebede

16 Jun 2026

Delayed Health-Seeking Behavior and Its Associated Factors among Cancer Patients in Ethiopia: A Systematic Review and Meta-Analysis, 2025

PONE-D-25-30632R3

Dear Dr. Abdulwehab

We’re pleased to inform you that your manuscript has been judged scientifically suitable for publication and will be formally accepted for publication once it meets all outstanding technical requirements.

Within one week, you’ll receive an e-mail detailing the required amendments. When these have been addressed, you’ll receive a formal acceptance letter and your manuscript will be scheduled for publication.

An invoice will be generated when your article is formally accepted. Please note, if your institution has a publishing partnership with PLOS and your article meets the relevant criteria, all or part of your publication costs will be covered. Please make sure your user information is up-to-date by logging into Editorial Manager at Editorial Manager® and clicking the ‘Update My Information' link at the top of the page. For questions related to billing, please contact billing support.

If your institution or institutions have a press office, please notify them about your upcoming paper to help maximize its impact. If they’ll be preparing press materials, please inform our press team as soon as possible -- no later than 48 hours after receiving the formal acceptance. Your manuscript will remain under strict press embargo until 2 pm Eastern Time on the date of publication. For more information, please contact onepress@plos.org.

Kind regards,

Adera Debella Kebede, MSC

Academic Editor

PLOS One

Additional Editor Comments (optional):

Reviewers' comments:

# Editor report: Acceptance letter

**DOI:** 10.1371/journal.pone.0352869.r008

**Authors:** Adera Debella Kebede, Adera Debella Kebede, Adera Debella Kebede, Adera Debella Kebede

PONE-D-25-30632R3

PLOS One

Dear Dr. Abdulwehab,

I'm pleased to inform you that your manuscript has been deemed suitable for publication in PLOS One. Congratulations! Your manuscript is now being handed over to our production team.

At this stage, our production department will prepare your paper for publication. This includes ensuring the following:

* All references, tables, and figures are properly cited

* All relevant supporting information is included in the manuscript submission,

* There are no issues that prevent the paper from being properly typeset

You will receive further instructions from the production team, including instructions on how to review your proof when it is ready. Please keep in mind that we are working through a large volume of accepted articles, so please give us a few days to review your paper and let you know the next and final steps.

Lastly, if your institution or institutions have a press office, please let them know about your upcoming paper now to help maximize its impact. If they'll be preparing press materials, please inform our press team within the next 48 hours. Your manuscript will remain under strict press embargo until 2 pm Eastern Time on the date of publication. For more information, please contact onepress@plos.org.

You will receive an invoice from PLOS for your publication fee after your manuscript has reached the completed accept phase. If you receive an email requesting payment before acceptance or for any other service, this may be a phishing scheme. Learn how to identify phishing emails and protect your accounts at https://explore.plos.org/phishing.

If we can help with anything else, please email us at customercare@plos.org.

Thank you for submitting your work to PLOS ONE and supporting open access.

Kind regards,

PLOS ONE Editorial Office Staff

on behalf of

Dr. Adera Debella Kebede

Academic Editor

PLOS One
